# The Secreted Acid Phosphatase Domain-Containing GRA44 from Toxoplasma gondii Is Required for c-Myc Induction in Infected Cells

**DOI:** 10.1128/mSphere.00877-19

**Published:** 2020-02-19

**Authors:** William J. Blakely, Michael J. Holmes, Gustavo Arrizabalaga

**Affiliations:** a Department of Pharmacology and Toxicology, Indiana University School of Medicine, Indianapolis, Indiana, USA; b Department of Biochemistry and Molecular Biology, Indiana University School of Medicine, Indianapolis, Indiana, USA; University of Georgia

**Keywords:** GRA44, MYR1, *Toxoplasma*, c-Myc, phosphatase, translocation

## Abstract

During host cell invasion, the eukaryotic pathogen Toxoplasma gondii forms a parasitophorous vacuole to safely reside within the cell, while it is partitioned from host cell defense mechanisms. From within this safe niche, parasites sabotage multiple host cell systems, including gene expression, apoptosis, and intracellular immune recognition, by secreting a large arsenal of effector proteins. Many parasite proteins studied for active host cell manipulative interactions have been kinases. The translocation of effectors from the parasitophorous vacuole into the host cell is mediated by a putative translocon complex, which includes the proteins MYR1, MYR2, and MYR3. Whether other proteins are involved in the structure or regulation of this putative translocon is not known. We have discovered that the secreted protein GRA44, which contains a putative acid phosphatase domain, interacts with members of this complex and is required for host cell effects downstream of effector secretion. We have determined that GRA44 is processed in a region with homology to sequences targeted by protozoan proteases of the secretory pathway and that both major cleavage fragments are secreted into the parasitophorous vacuole. Immunoprecipitation experiments showed that GRA44 interacts with a large number of secreted proteins, including MYR1. Importantly, conditional knockdown of GRA44 resulted in a lack of host cell c-Myc upregulation, which mimics the phenotype seen when members of the translocon complex are genetically disrupted. Thus, the putative acid phosphatase GRA44 is crucial for host cell alterations during *Toxoplasma* infection and is associated with the translocon complex which *Toxoplasma* relies upon for success as an intracellular pathogen.

**IMPORTANCE** Approximately one-third of humans are infected with the parasite Toxoplasma gondii. *Toxoplasma* infections can lead to severe disease in those with a compromised or suppressed immune system. Additionally, infections during pregnancy present a significant health risk to the developing fetus. Drugs that target this parasite are limited, have significant side effects, and do not target all disease stages. Thus, a thorough understanding of how the parasite propagates within a host is critical in the discovery of novel therapeutic targets. *Toxoplasma* replication requires that it enter the cells of the infected organism. In order to survive the environment inside a cell, *Toxoplasma* secretes a large repertoire of proteins, which hijack a number of important cellular functions. How these *Toxoplasma* proteins move from the parasite into the host cell is not well understood. Our work shows that the putative phosphatase GRA44 is part of a protein complex responsible for this process.

## Abstract (precis)

Approximately one-third of humans are infected with the parasite Toxoplasma gondii. *Toxoplasma* infections can lead to severe disease in those with a compromised or suppressed immune system. Additionally, infections during pregnancy present a significant health risk to the developing fetus. Drugs that target this parasite are limited, have significant side effects, and do not target all disease stages. Thus, a thorough understanding of how the parasite propagates within a host is critical in the discovery of novel therapeutic targets. *Toxoplasma* replication requires that it enter the cells of the infected organism. In order to survive the environment inside a cell, *Toxoplasma* secretes a large repertoire of proteins, which hijack a number of important cellular functions. How these *Toxoplasma* proteins move from the parasite into the host cell is not well understood. Our work shows that the putative phosphatase GRA44 is part of a protein complex responsible for this process.

## INTRODUCTION

Toxoplasma gondii is an obligate intracellular eukaryotic pathogen infecting an estimated one-third of the human population globally. Approximately 15% of the U.S. population is positive for *Toxoplasma* infection (1), while some countries in Europe and South America have much higher infection rates. Within the human host, *Toxoplasma* exists as either highly proliferative tachyzoites, which are responsible for the acute stage of the infection, or latent bradyzoite cysts, which form in various tissues and which establish a chronic infection. While most infections are asymptomatic, in immunocompromised individuals and lymphoma patients, new infections or reactivation of preexisting cysts can lead to toxoplasmic encephalitis, among other complications (2–4). Additionally, a primary infection puts pregnant women at risk of passing parasites to the developing fetus, which can cause miscarriage and severe birth defects (5).

Due to its biological niche as an obligate intracellular parasite, *Toxoplasma* depends upon remodeling the host cell environment to facilitate its own growth and survival. As the parasite invades a host cell, it forms an insular vacuole, known as the parasitophorous vacuole (PV), within which it may safely replicate undisturbed by host cell innate immune machinery. Within the PV, parasites interface with the host cell through the PV membrane (PVM) while avoiding direct contact with host cell components. Mediation of interactions between parasites and the host cell is accomplished by a multitude of parasite proteins that are secreted during invasion from the rhoptries (ROP proteins) and during intracellular division from the dense granules (GRA proteins). Many of these proteins are secreted beyond the PV into the host cell to directly manipulate host processes, like transcription, apoptosis, immune responses, and metabolism (6, 7). Additional proteins are secreted but are retained within the PV for the purpose of trafficking these effectors to the host cytoplasm. The proteins MYR1, MYR2, and MYR3, components of a putative translocon system, are secreted into the PV and are responsible for altering a wide array of host processes by trafficking effectors to the host (8). Secreted proteins, especially those with enzymatic activity, such as kinases and phosphatases, are of great interest, as they hold potential as drug targets due to their exclusivity to apicomplexans and importance to parasite survival. Two kinases secreted during invasion and intracellular growth that are known to be critical for host manipulation and parasite virulence are ROP16 (9) and ROP18 (10). The ROP16 kinase acts to downregulate STAT3/6 in the host nucleus, which results in altered transcription (11). ROP18, in partnership with GRA7, counteracts host cell immune responses by phosphorylation and inactivation of host immunity-related GTPase (IRG) proteins, which otherwise act to signal PV degradation (12). Other *Toxoplasma* kinases known to be secreted into the host cell include WNG1 and WNG2, formerly ROP35 and ROP34, respectively (13). Additionally, several pseudokinases have been shown to be secreted into the host and are implicated in host cell manipulation, such as ROP5, which complexes with ROP18, conferring binding affinity to host IRGA6 (12).

Whether secreted phosphatases play a role similarly as important as that of secreted kinases and pseudokinases remains largely unexplored. The secreted phosphatase PP2C has been proposed to reduce the apoptosis of infected host cells (14), and the phosphatase PP2C-hn has been found in the host nucleus (15), although its function remains unclear. To expand our understanding of phosphatases secreted into either the host cell or PV, we bioinformatically identified 32 proteins predicted to have both a phosphatase domain and a signal sequence. Of those identified, we characterized the biological role of TGGT1_228170, which was previously identified to be part of the inner membrane complex and which was named IMC2A by Mann and Beckers (16). However, contrary to the initially documented localization, this protein contains characteristics of secreted proteins, namely, a signal sequence and predicted *Toxoplasma* export element (TEXEL) motifs, protease cleavage sites found in many *Toxoplasma* secreted proteins (17). Here, we show that this protein is both processed and secreted into the PV, where it interacts with the proposed translocation complex of MYR1/2/3. Importantly, we show that TGGT1_228170, now renamed GRA44, is critical for activation of the host oncogenic factor c-Myc.

## RESULTS

### Bioinformatic search for secreted phosphatase.

**FIG 1:**
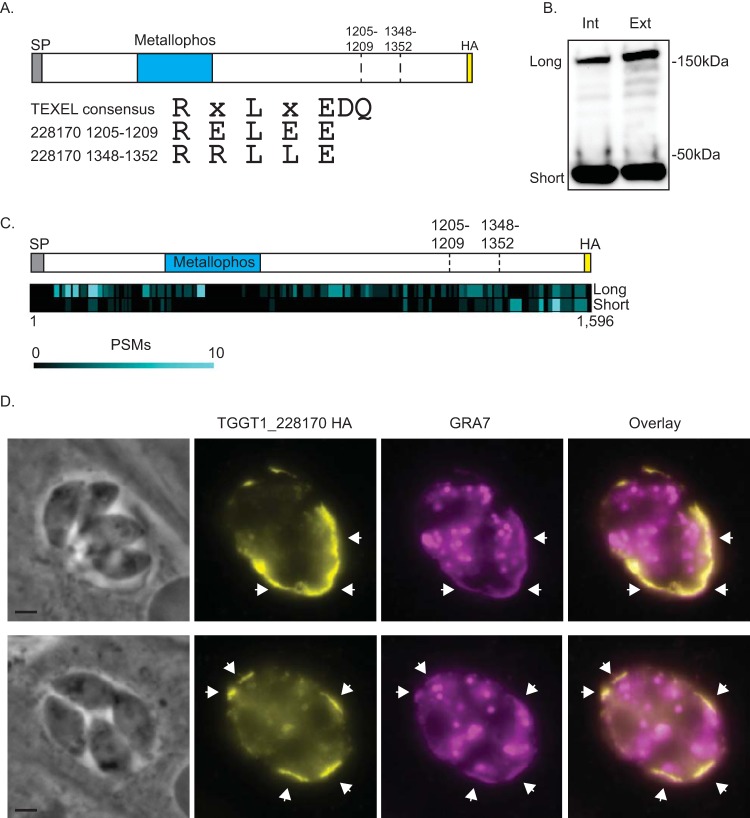
TGGT1_228170 is processed and secreted into the parasitophorous vacuole. To determine the localization of TGGT1_228170, we introduced a C-terminal HA epitope tag in the endogenous gene. (A) Schematic showing the relative positions of the signal peptide (SP), the phosphatase domain (Metallophos), and the two putative TEXEL cleavage sites at amino acids 1205 to 1209 and amino acids 1348 to 1352. The C-terminal HA epitope tag is also shown. Below the schematic are the sequences of the TEXEL1 and TEXEL2 cleavage domains compared to the consensus sequence of the TEXEL domain. (B) Western blot of protein lysates from intracellular (Int) and extracellular (Ext) parasites of the TGGT1_228170(HA)-expressing strain probed with antibodies against HA. Two stable forms, labeled long and short, are detected. (C) Heat map illustrating the relative positions of peptide-to-spectrum matches (PSMs) from mass spectrometric analysis of the long and short forms of TGGT1_228170(HA) in respect to the full protein and the putative cleavage sites. (D) Immunofluorescence assay (IFA) of intracellular parasites of the IFA images of strain expressing TGGT1_228170(HA) stained for the parasitophorous vacuole protein GRA7 (in magenta) and for HA (in yellow). Bars = 2 μm.

To begin identifying putative secreted phosphatases, we used a bioinformatics approach starting with all proteins annotated in the ToxoDB Toxoplasma gondii genome database (toxodb.org). A search of all *Toxoplasma* genes with the BLAST program, filtered by including only genes whose products contain predicted phosphatase domains and signal peptides, generated a list containing 32 proteins of potential interest (see Table S1 in supplemental material). To prioritize our studies on phosphatases that are likely to play an important role in parasite propagation, we ranked our list according to the gene fitness scores assigned through a genome-wide CRISPR/Cas9 knockout study (18). Among these was TGGT1_228170, a protein that contains a predicted acid phosphatase domain (Fig. 1A) and, that despite having a signal sequence, was previously described as localizing to the inner membrane complex (IMC) (16). However, multiple lines of evidence suggest that it may indeed be secreted. First, the homologous protein UIS2 in the related apicomplexan parasite Plasmodium berghei has been shown to have a secreted ortholog (Pf3D7_1464600) in Plasmodium falciparum (19). Second, TGGT1_228170 has been repeatedly detected in BioID experiments as an interactor of proteins localized to the PV lumen and PV membrane microenvironments (20). Finally, analysis of the protein sequence revealed multiple putative *Toxoplasma* export elements (TEXEL), and at the time that this investigation began, the sequence was defined as RXLXD/E (21) and has since been refined as RRL (22). TEXEL sequences are recognized by a Golgi apparatus-associated protease, aspartyl protease V (ASP5), which cleaves proteins as part of the secretory pathway to the PV/PV membrane (PVM) and host cell. Based on these criteria and the fact that TGGT1_228170 was assigned a gene knockout fitness score of −3.28 (18), indicating that it substantially contributes to parasite fitness, we decided to revisit the localization and function of this protein.

10.1128/mSphere.00877-19.4 TABLE S1 Proteins annotated as phosphatases and predicted to contain a signal peptide. The fitness score is the mean phenotype score based on a genome-wide CRISPR screen (15). Download Table S1, PDF file, 0.04 MB. Copyright © 2020 Blakely et al. 2020 Blakely et al. This content is distributed under the terms of the Creative Commons Attribution 4.0 International license.

### TGGT1_228170 is processed and secreted into the parasitophorous vacuole.

To determine the localization of TGGT1_228170, we introduced sequences encoding three C-terminal hemagglutinin (HA) epitope tags (3×HA) into the endogenous gene by homologous recombination (Fig. 1A). Western blot analysis of protein extract from both intracellular and extracellular parasites of the TGGT1_228170(HA) line showed a band of approximately 180 kDa, which is the expected size for the full protein (Fig. 1B). However, a second prominent band at approximately 40 kDa was also noted in both intracellular and extracellular parasites (Fig. 1B). This second, smaller band is consistent with processing at either of two areas with homology to TEXEL sites (Fig. 1A). The sequence for the first of these putative cleavage sites is RELEE (amino acids 1205 to 1209), which is consistent with the previous TEXEL consensus sequence, while the sequence for the second is RRLLE (amino acids 1348 to 1352), which is consistent with the RRL consensus sequence (Fig. 1A).

To confirm the identity of the 40-kDa fragment observed in the Western blot, endogenously tagged TGGT1_228170 was immunoprecipitated and the eluate was separated by SDS-PAGE. Both the 180-kDa (long) and 40-kDa (short) bands were excised from the PAGE gel and analyzed separately by mass spectrometry (MS). The results confirmed that both bands corresponded to TGGT1_228170. For the long form, we detected 192 peptides distributed throughout the full protein sequence (Fig. 1C). MS analysis of the band migrating to 40 kDa revealed 74 peptides corresponding to TGGT1_228170, and 60 of these were located after the second putative cleavage site (Fig. 1C). Thus, TGGT1_228170 is processed, and both the full-length and C-terminal forms are stable.

Finally, to determine the localization of TGGT1_228170, we performed immunofluorescence assays (IFAs) of TGGT1_228170(HA) parasites. Consistent with the presence of a signal sequence and putative TEXEL sites, TGGT1_228170 was detected within the PV lumen and at the PV membrane (PVM) (Fig. 1D). Based on this localization and corroborative findings by Coffey et al. for the same protein (13), TGGT1_228170 should be designated a GRA protein, and we henceforth refer to it as GRA44.

### Amino acids 1348 to 1352 are required for efficient processing of GRA44.

**FIG 2:**
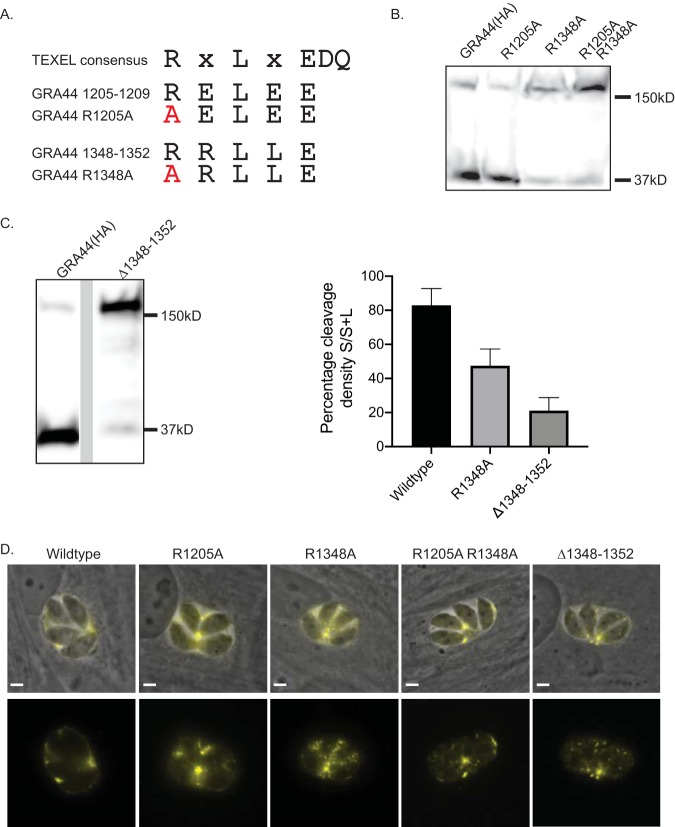
The second putative TEXEL motif is critical for efficient processing of GRA44. To determine the site responsible for the processing of GRA44, we expressed exogenous copies of GRA44(HA) in which the first arginine of the putative cleavage sites was mutated to alanine individually (R1205A and R1348A) or in combination (R1205A/R1348A) or in which the 5 amino acids of the second putative site were deleted (Δ1348–1352). (A) Diagram of the TEXEL consensus sequence and the putative sites in GRA44, with the mutated amino acids indicated in red. (B) Western blot of lysates from parasites expressing the exogenous GRA44(HA), the R1205A or R1348A mutant version, or the double R1205A/R1348A mutant probed for HA. (C) (Left) Western blot of lysates from parasites expressing an exogenous copy of GRA44 lacking the second site (the Δ1348–1352 mutant) probed with antibodies against the HA epitope tag. (Right) Percent cleavage in the wild-type, R1205A, and Δ1348–1352 strains, which was determined by calculating the ratio of the density of the large band (L) over the sum of the density of both bands (S+L). The results for all data sets (*n* = 3, mean ± SD) were significantly different from those for the others, based on one-way analysis of variance (*P* < 0.05). (D) Representative images of intracellular parasites expressing each of the four GRA44 processing mutants. Images are of the immunofluorescence signal from the HA tag (in yellow) and of the HA signal overlaid on the phase image. Bars = 2 μm.

We hypothesized that the small C-terminal fragment of GRA44 detected by Western blotting and MS is the product of cleavage at either of the two putative TEXEL sequences. To investigate which of the two sites is actively cleaved, we exogenously expressed GRA44 in which the first arginine of either or both of these sites was mutated to alanine (Fig. 2A). The first arginine of TEXEL sites has previously been shown to be important for cleavage (21). Western blot analysis showed that mutating the first arginine of the first putative site (GRA44 R1205A) did not affect processing of the protein (Fig. 2B). In contrast, mutating the arginine in the second site (GRA44 R1348A) significantly reduced processing (Fig. 2B). The same result was observed when both putative TEXEL sites were mutated (GRA44 R1205A/R1348A) (Fig. 2B). Densitometry analysis showed that while for the wild-type protein 82.9% ± 9.9% (*n* = 3) of the total protein was cleaved, the cleavage in GRA44 R1348A was 47.4% ± 9.9% (*n* = 3) (Fig. 2C). Thus, it appears that the second TEXEL is the site for processing of GRA44, and it is referred to as the GRA44 TEXEL from here on. For a thorough examination of the identified TEXEL, we generated parasites exogenously expressing GRA44 in which either L1350 or E1352 was mutated to alanine (Fig. S1A). As was the case for the R1348A mutation, changing the central leucine in the GRA44 TEXEL to an alanine disrupted processing (Fig. S1B); however, mutant E1352A showed similar levels of processing as the wild-type protein (Fig. S1B).

10.1128/mSphere.00877-19.1 FIG S1 Mutational analysis of TEXEL2. (A) Alignment of the TEXEL consensus sequence and the GRA44 TEXEL sequence along with the sequence of the three mutant versions, the R1348A, L1350A, and E1352A mutants. (B) Western blot of lysates from parasites expressing exogenous wild-type or mutant GRA44 probed with HA antibodies. (C) Representative IFA images of intracellular parasites expressing each of the four mutant GRA44 proteins. Images are overlays of the phase and HA signal (in green). Download FIG S1, PDF file, 1.2 MB. Copyright © 2020 Blakely et al. 2020 Blakely et al. This content is distributed under the terms of the Creative Commons Attribution 4.0 International license.

Although we observed a significant reduction in cleavage after altering single amino acids in the GRA44 TEXEL, there appeared to be some residual C-terminal cleavage product present with all mutants (Fig. 2B and Fig. S1B). To ascertain whether this was the effect of persistent cleavage at TEXEL, despite the mutations, or cleavage at an alternative site, we generated an exogenously expressed GRA44 mutant in which all 5 amino acids that make up this TEXEL (amino acids 1348 to 1352) were deleted (the GRA44 Δ1348–1352 mutant). As expected, deletion of the TEXEL resulted in a significant loss of the C-terminal cleavage product (Fig. 2C). However, it was evident that there still remained a measurable amount being cleaved. The cleavage level for the Δ1348–1352 mutant was calculated to be 21.1 ± 7.7 (*n* = 3), which was significantly lower than that for the R1348A point mutant (Fig. 2C). Since the TEXEL was not present in this mutant form of GRA44, it is plausible that a cryptic site, potentially amino acids 1205 to 1209, was used. Alternatively, GRA44 may be cleaved by a TEXEL/ASP5-independent mechanism.

To determine whether effective processing is needed for the localization of GRA44 to the PV, we performed IFAs with parasites expressing each of the four mutant GRA44 proteins (the R1205A, R1348A, R1205A/R1348A, and Δ1348–1352 mutants). Interestingly, none of the mutations affected secretion and localization to the parasitophorous vacuole (Fig. 2D). Similarly, mutating L1350 or E1352 within the confirmed TEXEL site did not affect the PV localization of GRA44 (Fig. S1C). These results indicate that complete processing is not required for protein secretion.

### Both the N-terminal and C-terminal GRA44 cleavage products localize within the PV.

**FIG 3:**
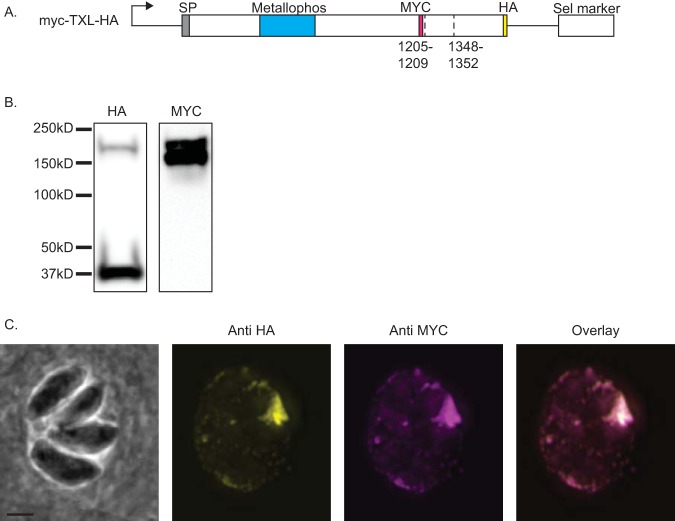
The GRA44 N-terminal cleavage product is secreted. To determine the stability and localization of the GRA44 N-terminal cleavage product, we expressed an exogenous copy of GRA44 with an internal MYC epitope tag and a C-terminal HA epitope tag. (A) Schematic of the exogenous GRA44 construct MYC-TXL-HA showing the positions of the MYC and HA epitope tags relative to the two putative cleavage sites. (B) Western blot of the MYC-TXL-HA parasite line separately probed with anti-HA and anti-MYC antibodies. (C) IFA images of MYC-TXL-HA-expressing parasites probed for HA (yellow) and MYC (magenta). Bar = 2 μm.

As the localization analysis performed depended on a C-terminal HA epitope tag and GRA44 is cleaved at an internal TEXEL site, we could detect only the full-length uncleaved protein and a smaller C-terminal fragment. Consequently, with the C-terminal HA-tagged protein, we could not determine the localization of the N-terminal cleavage product, which contains the putative acid phosphatase domain. Accordingly, we engineered a strain exogenously expressing GRA44 containing a MYC epitope tag inserted between amino acids 1203 and 1204, in addition to the HA epitope tag at the C terminus (Fig. 3A). Protein extracts from parasites expressing the dually tagged GRA44 were analyzed by Western blotting and probed separately with antibodies against the MYC or HA epitope (Fig. 3B). Probing of anti-MYC uncovered a band at approximately 140 kDa, in addition to the 180-kDa full-length protein (Fig. 3B). This 140 kDa correlates to the expected N-terminal end of GRA44 postcleavage. As observed previously, probing with antibodies against the HA epitope revealed the full-length GRA44 and the C-terminal fragment. Having established a parasite line that allows monitoring of two postprocessing fragments of GRA44, we investigated their respective localization by IFA. Regardless of whether we used HA or MYC antibodies, the protein was localized primarily to the PV, which suggests that the two major fragments, including the one containing the phosphatase domain, are secreted into the parasitophorous vacuole (Fig. 3C).

### Loss of GRA44 negatively affects parasite propagation.

**FIG 4:**
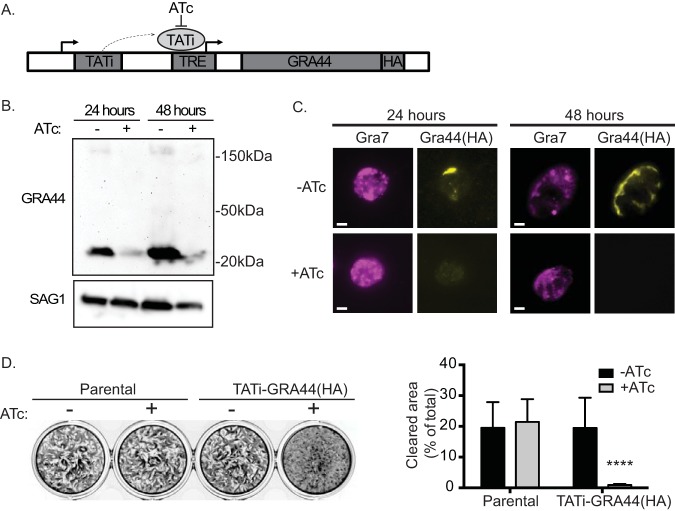
Knockdown of GRA44 disrupts parasite propagation. To determine the function of GRA44, we applied a tetracycline (Tet)-repressible system to establish a conditional GRA44 knockdown strain. (A) Diagram of the strategy used to generate the conditional knockdown of GRA44, as outlined in the Materials and Methods section. Sel marker, selective marker. (B) Quantitative Western blot of the TATi-GRA44(HA) parasite strain grown for either 24 or 48 h in the absence (lanes −) or the presence (lanes +) of ATc probed with HA to detect GRA44 and SAG1 as a loading control. (C) The reduction in GRA44 expression in the presence of ATc was confirmed by IFA of intracellular parasites of the TATi-GRA44(HA) strain grown with and without ATc for 24 or 48 h and probed with anti-HA antibodies. Bars = 2 μm. (D) Plaque assays were performed with the GRA44(HA) parasites (parental) or the TATi-GRA44(HA) parasites grown without (−) or with (+) ATc for 5 days. (Left) Representative results of a plaque assay. (Right) The results were quantitated based on the percentage of the cell monolayer cleared by the parasite (cleared area) and the average for biological and experimental triplicates (*n* = 3; mean ± SD; *P* < 0.0001, unpaired *t* test).

Through a genome-wide CRISPR screen, GRA44 was assigned a log_2_ relative fitness score of −3.28 (18), which would suggest that the loss of GRA44 would be a significant detriment to parasite propagation. Accordingly, we applied a tetracycline (Tet)-repressible system (23, 24) to establish a conditional GRA44 knockdown strain. Specifically, we used CRISPR to introduce a cassette encoding a drug-selective marker, a transactivator (TATi) protein, and a tetracycline response element (TRE) just upstream of the endogenous GRA44 start codon (Fig. 4A). To be able to monitor GRA44 protein expression, we engineered the conditional knockdown mutant using the GRA44(HA) strain. The resulting strain, TATi-GRA44(HA), was grown for 24 and 48 h in the absence and presence of the tetracycline analog anhydrotetracycline (ATc), and GRA44 expression was monitored by Western blotting (Fig. 4B) and IFA (Fig. 4C). At both time points, protein levels were significantly reduced in the presence of ATc when observed by either Western blotting or IFA (Fig. 4B and C). Thus, we successfully established a strain in which the expression of GRA44 could be conditionally turned down.

Interestingly, we noted that the small C-terminal fragment from the TATi-GRA44(HA) strain was smaller than what is observed with the parental endogenous HA-tagged strain (Fig. 1B and 4B). Sequencing of GRA44 in the TATi-GRA44(HA) strain showed that a 315-bp fragment, which encodes the last 105 amino acids, was deleted, leaving the HA tag in frame. Surprisingly, we did not note any significant propagation defect and successfully complemented the strain with a full-length gene construct (Fig. 4D; compare the results for the parental strain to those for the knockdown strain grown without ATc). Thus, deletion of that region does not affect localization or function. Regardless, this strain allows us to study the consequence of eliminating GRA44 expression upon ATc addition. In addition, to ensure that any phenotype is due to the downregulation of GRA44, we complemented this strain with the addition of a wild-type copy of the gene (see below). Importantly, when the TATi-GRA44(HA) strain was grown in the presence of ATc, depleting GRA44, propagation was significantly affected compared to the propagation by the same strain under normal conditions (Fig. 4D). For the conditional knockdown strain, in the absence of ATc, we quantitated an average cell clearance of 19.4% ± 9.9%, which was reduced to 0.9% ± 0.4% when ATc was included in the growth medium (Fig. 4D). Therefore, the conditional knockdown of GRA44 significantly reduces parasite propagation in tissue culture.

### Processing is not necessary for GRA44 function.

**FIG 5:**
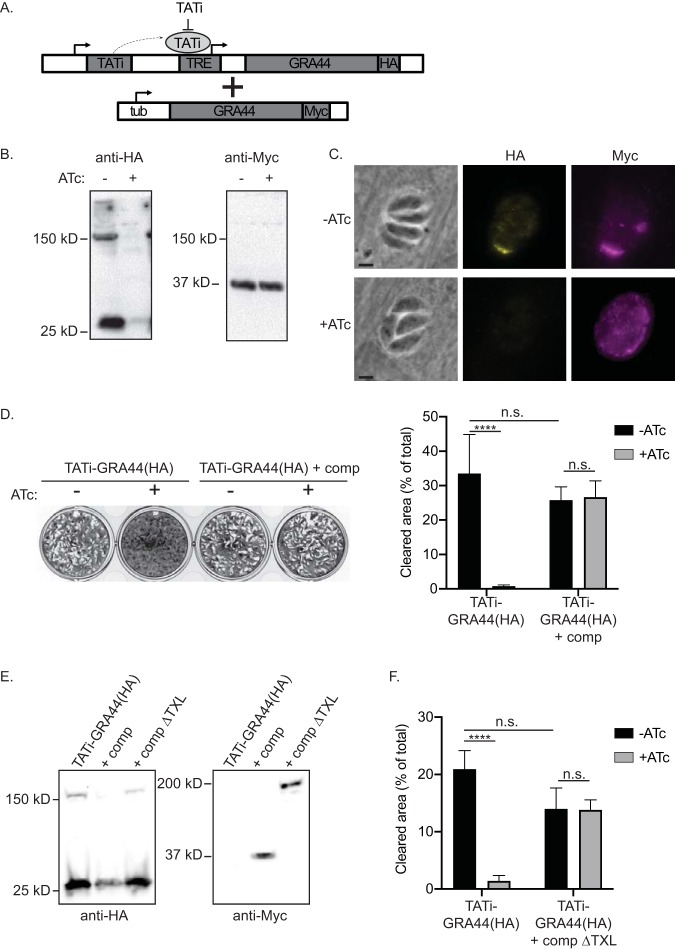
The phenotype of GRA44 knockdown can be complemented. To establish a direct connection between the lack of GRA44 and the phenotype observed, we established a complemented strain by adding an exogenous copy of wild-type GRA44 to the TATi-GRA44(HA) conditional knockdown strain. (A) Diagram of the strategy used to establish a complemented strain, TATi-GRA44(HA)comp. The wild-type copy of GRA44 added to the TATi-GRA44(HA) strain contains a C-terminal MYC epitope tag and is driven by the *Toxoplasma* tubulin promoter (tub). (B) Western blot of lysates from the TATi-GRA44(HA)comp parasites grown with and without ATc for 48 h. The blots were probed for HA and MYC. (C) Representative IFA images of TATi-GRA44(HA)comp with and without ATc treatment stained for the HA-tagged regulatable GRA44 and the MYC-tagged constitutive exogenous copy. Bars = 2 μm. (D) (Left) Plaque assays were performed with the knockdown [TATi-GRA44(HA)] and the complemented [TATi-GRA44(HA)comp] strains grown without (−) or with (+) ATc for 5 days. (Right) The average percentage of the cell monolayer cleared from biological and experimental triplicates is shown on a bar graph (*n* = 3; mean ± SD; ****, *P* < 0.0001; n.s., not significant [significance was determined by one-way analysis of variance, followed by Tukey’s test]). (E) TATi-GRA44(HA) was complemented with an exogenous copy of GRA44 containing TEXEL with a deletion of amino acids 1348 to 1352 and a C-terminal MYC tag [TATi-GRA44(HA)compΔTXL]. Lysates from TATi-GRA44(HA), wild-type complemented strain TATi-GRA44(HA)comp, and ΔTXL complemented strain TATi-GRA44(HA)compΔTXL were analyzed by Western blotting and probed for HA and MYC. (F) Plaque assays were performed with the TATi-GRA44(HA) and TATi-GRA44(HA)compΔTXL strains. Parasites were grown 5 days without (−) or with (+) ATc. The average percentage of the cell monolayer cleared for biological and experimental triplicates is shown on a bar graph (*n* = 3; mean ± SD; ****, *P* < 0.0001; n.s., not significant [significance was determined by one-way analysis of variance, followed by Tukey’s test]).

To confirm that the propagation defect observed upon knockdown of GRA44 was due to the reduction of GRA44 levels, we tested whether an exogenous copy of GRA44 could complement the phenotype. For this purpose, we introduced a copy of GRA44 with a C-terminal MYC epitope tag into the TATi-GRA44(HA) strain to generate a complemented strain, TATi-GRA44(HA)comp, (Fig. 5A). The exogenous copy of GRA44(MYC) was processed and secreted as expected (Fig. 5B and C). In the absence of ATc, both the endogenous HA-tagged GRA44 and the exogenous MYC-tagged GRA44 were detected in this strain by both Western blotting and IFA (Fig. 5B and C). As expected, addition of ATc resulted in the knockdown of GRA44(HA) but not that of the exogenous GRA44(MYC) (Fig. 5B and C). Plaque assays of both the knockdown [TATi-GRA44(HA)] and complemented [TATi-GRA44(HA)comp] strains with and without ATc were performed in parallel to determine the ability of GRA44 to complement the phenotype. Consistent with the previous result, addition of ATc to the TATi-GRA44(HA) strain severely impaired plaque formation (Fig. 5D). Importantly, the presence of a constitutively expressed copy of GRA44 complemented this phenotype (Fig. 5D). While the percent clearance of the host cell in the presence of ATc was 0.8% ± 0.4% for the knockdown strain after 5 days in culture, it was 26.6% ± 3.7% for the complemented strain, which was statistically equal to what was observed without ATc with either strain (Fig. 5D). Complementation of the plaque formation phenotype by the addition of a wild-type copy confirms that GRA44 is critical for the efficient propagation of *Toxoplasma*.

Having established complementation of the propagation phenotype, we set out to determine whether processing of GRA44 was needed for function. Accordingly, we complemented the TATi-GRA44(HA) strain with an exogenous copy of GRA44(MYC) containing a TEXEL deletion of residues 1348 to 1352 to obtain the strain GRA44compΔTXL. For the GRA44compΔTXL strain under normal growth conditions without ATc, endogenous GRA44(HA) was detected by Western blotting at a size similar to that in the TATi-GRA44 and TATi-GRA44comp strains; however, the exogenous MYC-tagged GRA44compΔTXL copy was seen as a mostly uncleaved form of the protein, in contrast to that in the wild-type complemented strain, which was processed (Fig. 5E). Remarkably, the GRA44compΔTXL complemented parasite strain was no longer sensitive to the presence of ATc (Fig. 5E). These results indicate that the complete cleavage of GRA44 is not necessary for function.

### GRA44 interacts with members of the effector translocation complex.

Our results have thus far shown GRA44 to be of significant importance for successful parasite propagation; however, the specific function of this protein within the parasitophorous vacuole is unclear. To shed light on its function, we examined what proteins interact with GRA44 by developing a comprehensive interactome. For this purpose, the GRA44 protein was immunoprecipitated from the HA-tagged GRA44(HA) line and coprecipitating peptides were analyzed by mass spectrometry. After three replicate experiments and control assays with nonspecific beads, the data were statistically analyzed by significant analysis of interactome (SAINT) (25) computational predictive analysis (Data Set S1). With a SAINT score of >0.8 used as a cutoff, we obtained a list of 35 putative interactors, of which 8 were ribosomal and snRNP proteins and likely to be nonspecific (Table S2). Significantly, of the remaining 27 putative interactors, 23 had predicted signal peptides, which indicates that they are likely secreted proteins (Table 1). Among these were eight known GRA proteins (GRA9, -16, -25, -33, -34, -45, -50, and -52); the parasitophorous vacuole membrane-associated protein MAF1; and MYR1, a known member of the effector translocation complex (26, 27).

**TABLE 1:** Putative GRA44 interactors identified by IP^*a*^

Protein	Product description
TGGT1_316250	GRA45
TGGT1_204340	GRA54
TGGT1_254470	MYR1
TGGT1_319340	GRA52
TGGT1_279100	MAF1a
TGGT1_251540	GRA9
TGGT1_203600	GRA50
TGGT1_304955	Serine/threonine-specific protein phosphatase (PPM11C)
TGGT1_315610	Hypothetical protein
TGGT1_203290	GRA34
TGGT1_270320	Protein phosphatase 2C domain-containing protein (PPM3C)
TGGT1_258870	Hypothetical protein
TGGT1_311720	Chaperonin protein BiP
TGGT1_226240	Putative bud site selection protein
TGGT1_216770	Hypothetical protein
TGGT1_220950	MAF1b
TGGT1_270240	MAG1
TGGT1_200360	Hypothetical protein
TGGT1_290700	GRA25
TGGT1_258458	Hypothetical protein
TGGT1_262050	Rhoptry kinase family protein ROP39
TGGT1_410360	MAF1 copy
TGGT1_247440	GRA33
TGGT1_229480	Putative calcium binding protein precursor
TGGT1_208830	GRA16
TGGT1_410370	MAF1 copy

a The criteria used were a SAINT score (for the signal peptide) of >0.8 and not being a ribosomal protein (TGGT1_309820, TGGT1_207840, TGGT1_266070, TGGT1_248480). The shaded proteins have a predicted signal peptide based on SignalP analysis. Proteins are listed on the basis of their SAINT scores, total peptides, and the fold change in the number of peptides in experimental IPs over that for the controls (see Table S2 in the supplemental material).

10.1128/mSphere.00877-19.5 TABLE S2 Proteins identified through immunoprecipitation and MS/MS with a SAINT score of 0.8 or higher. The numbers of peptides shown are the total for all three experiments or controls, and the fold change is the number of peptides in experimental IPs over that in control ones. Download Table S2, PDF file, 0.1 MB. Copyright © 2020 Blakely et al. 2020 Blakely et al. This content is distributed under the terms of the Creative Commons Attribution 4.0 International license.

10.1128/mSphere.00877-19.7 DATA SET S1 Mass spectroscopy result of immunoprecipitation of GRA44. Download Data Set S1, PDF file, 0.1 MB. Copyright © 2020 Blakely et al. 2020 Blakely et al. This content is distributed under the terms of the Creative Commons Attribution 4.0 International license.

**FIG 6:**
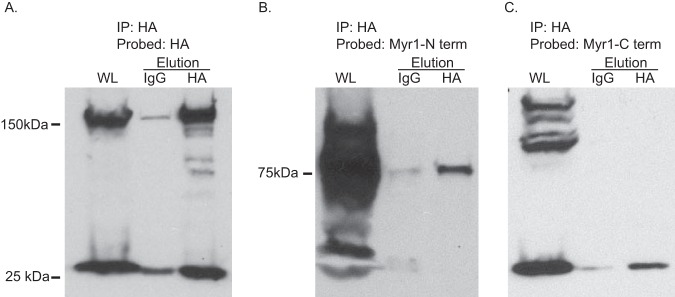
GRA44 interacts with MYR1. To confirm the interaction between GRA44 and MYR1, we performed immunoprecipitation from GRA44(HA)-expressing parasites and probed for GRA44(HA) (A) or either the N terminus (B) or the C terminus (C) of MYR1 by Western blotting. As controls, we performed the immunoprecipitation with IgG-conjugated beads. In all blots, the first lane is whole lysate (WL), the middle lane is the eluate from IgG beads, and the last lane is the eluate from the HA beads.

To confirm the interaction with MYR1, for which there are antibodies (26), we performed coimmunoprecipitation (co-IP) assays. Purified lysate was precipitated from GRA44(HA) parasites on mouse anti-HA beads, and the eluates were evaluated by Western blotting for the presence of GRA44 and MYR1. For MYR1, which is also processed by ASP5 at a TEXEL site, we probed with antibodies for either the C-terminal or the N-terminal cleavage product. Immunoprecipitated GRA44 yielded a significant amount of either MYR1 fragment compared to that for the control IgG bead eluate of the same source (Fig. 6). Thus, GRA44 appears to interact with a member of the effector translocation system.

### GRA44 is required for c-Myc induction.

**FIG 7:**
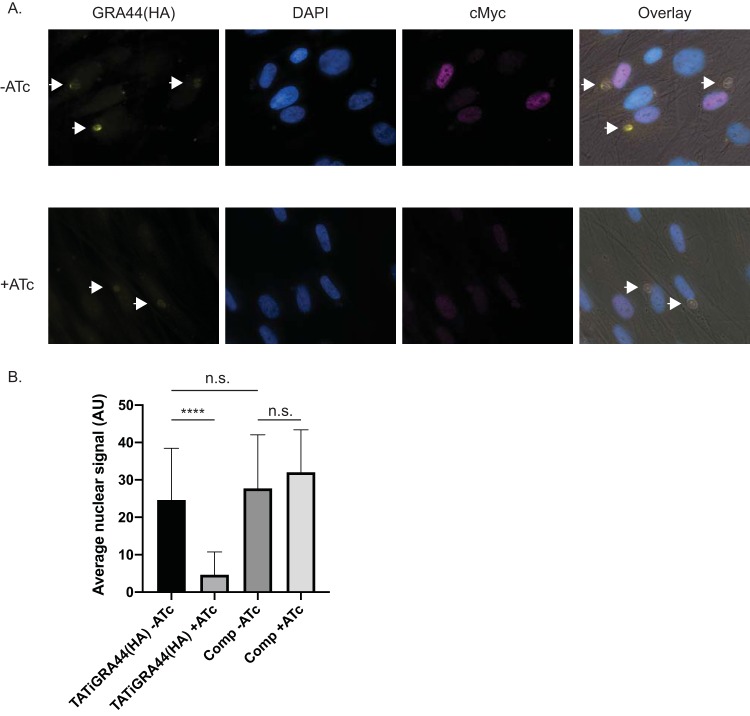
c-Myc activation in the GRA44 mutant strain. To assess the host cell response upon GRA44 knockdown, host nuclear c-Myc expression was quantified by fluorescence microscopy after invasion by either the knockdown TATi-GRA44(HA) or the complemented knockdown TATi-GRA44(HA)comp parasite strain in the presence and the absence of ATc. (A) Representative immunofluorescence images of TATI-GRA44(HA) parasites grown without ATc (−ATc) or with ATc (+ATc). The cultures were stained for HA to detect GRA44 (yellow), DAPI to detect DNA (blue), and host c-Myc (magenta). Arrows point at vacuoles with more than 2 parasites. (B) The graph represents the average quantified nuclear signal from c-Myc antibody staining across biological and experimental triplicates. Arbitrary units (AU) were used in comparing the nuclear c-Myc signal intensity (*n* = 3; ****, *P* < 0.0001; n.s., not significant [significance was determined by one-way analysis of variance, followed by Tukey’s test]).

MYR1 was identified through a forward genetic screen to be required for the translocation of parasite effectors, such as GRA16 and GRA24, and the ensuing upregulation of the host cell oncogene c-Myc (26). Given GRA44’s interaction with MYR1, we tested whether it might be involved in the same functions, specifically, c-Myc induction. For this purpose, TATi-GRA44(HA) parasites were grown for 24 h either with or without ATc, released from the host cells, and allowed to infect new cells. Those that came from the conditions with ATc were kept in ATc, while those from the culture without ATc were kept without it. After 12 h of growth, the cultures were fixed and an IFA for human c-Myc was performed (Fig. 7). Images obtained by merged phase-contrast microscopy and HA, MYC, DAPI (4′,6-diamidino-2-phenylindole), and c-Myc staining were used to locate host cells infected by single PVs with greater than 1 parasite per vacuole, and single-channel images of c-Myc staining within these host nuclei boundaries were quantitated with ImageJ software. Addition of ATc to TATi-GRA44(HA) parasites produced a significantly reduced level of GRA44 (Fig. 7A), consistent with the results observed by Western blotting, and, importantly, reduced the c-Myc signal by approximately 5-fold, a significantly dampened response compared to that seen under normal conditions with the same strain (Fig. 7B). No significant difference in the c-Myc signal was observed in the complemented strain upon ATc addition.

## DISCUSSION

As part of its life cycle, *Toxoplasma* invades and dwells within host cells, where it utilizes nutrient resources readily available within a host. Since residence inside a host is key to parasite survival and propagation, *Toxoplasma* parasites have multiple means of altering the status of their host to better suit their needs. Examples of the changes induced within the host include apoptosis inhibition (14), innate immune system disruption (28), host cytoskeleton restructuring (29), and global changes to host gene transcription (9). These critical host cell alterations are accomplished by a large arsenal of parasite effectors that are secreted into the host cell during invasion and intracellular division. Secretion of these effectors involves highly coordinated actions by two unique parasite organelles: the rhoptries and the dense granules. The rhoptries discharge proteins known as ROPs during invasion, while the dense granules release their contents into the PV during intracellular division. While some of these so-called GRA proteins remain within the PV space or associate with the PV membrane (PVM), many of them are translocated across the PVM into the host, where they affect numerous signaling pathways. How these proteins move from the PV to the host is becoming clear with the discovery of proteins that appear to form part of a putative translocon (24). The work presented here shows that GRA44 (TGGT1_228170), previously known as IMC2A (16), interacts with members of this complex and that it is essential for some of the host cell events downstream of effector translocation.

Work from Coffey et al. identified GRA44 to be a substrate of ASP5 through a comparative proteomic approach (13). They showed that, indeed, GRA44 is processed and secreted into the PV, which we have corroborated with the work presented here. Their analysis of GRA44 identified two cleavage sites, one at positions 83 to 85 and another at positions 1348 to 1350. Nonetheless, their studies did not determine whether all cleavage products were secreted or the function/role of GRA44 within the PV. Here, we report that the two major GRA44 products are secreted into the PV. This is important, as it is the first evidence that the fragment containing the putative acid phosphatase is indeed in the PV. In our study, we show that the processing is not necessary for either the secretion or the function of GRA44. This is consistent with the fact that other secreted proteins containing TEXEL motifs, such as MYR1 and WNG1/2, can still be secreted in the absence of ASP5 activity (13). Fortuitously, through a spontaneous deletion in the knockdown strain, we also determined that the last 105 amino acids of the protein are not needed for function or localization.

Proteomic analysis of proteins that coimmunoprecipitated with GRA44 revealed interactions with known secreted proteins, including numerous GRAs. Among these interactors are GRA9, part of the intravacuolar tubular network (30); GRA16, an effector altering the host cell cycle through the p53 pathway (31); GRA25, a macrophage-dependent immune modulator (32); and GRA33, GRA34, GRA45, GRA50, GRA52, and members of the multicopy mitochondrion association factor 1 (MAF1) family. MAF1 is involved in recruiting the host mitochondria to the PV membrane surface (33). Most importantly, immunoprecipitation (IP) revealed an interaction between MYR1 and GRA44, which we independently confirmed by Western blotting (Fig. 6). Interestingly, as we were studying the interactome of GRA44, we learned of ongoing work in John C. Boothroyd’s lab showing a physical and functional interaction between MYR1 and GRA44 (34). MYR1 was initially identified through a forward genetic screen for *Toxoplasma* mutants unable to activate host c-Myc and translocate effectors, such as GRA16 and GRA24 (22). Further screening for such mutants identified MYR2 and MYR3 to also be required for effector translocation (27). Thus, MYR1/2/3 appear to be part of a putative translocon complex, although only MYR1 and MYR3 have been confirmed to directly interact (27). MYR1-dependent effectors are responsible for driving a broad range of host cell effects early on during infection. MYR1-dependent host responses include the upregulation of E2F transcription factors and the downregulation of interferon signaling (8). The interaction between GRA44 and MYR1 would suggest a function in translocation for this putative acid phosphatase.

One of the challenges in determining the relevance of interactions among dense granule proteins is that those interactions could be within the dense granules or during transit and not necessarily once in the PV or host, where they exert their function. Nonetheless, our data strongly suggest that the interaction with MYR1 is functionally relevant. Similar to the results obtained after the knockout of MYR1, the knockdown of GRA44 resulted in a significant reduction of c-Myc activation. Together, these findings support the notion that GRA44 is a member of the MYR1/2/3 translocon machinery. Whether its role in this process is structural or regulatory remains unknown and would require further investigation. The possibility that GRA44 plays a regulatory function is suggested by the presence of a putative catalytic site reminiscent of acid phosphatases. Whether GRA44 is an active phosphatase or a pseudophosphatase remains to be determined.

In the biology of animals, plants, and fungi, acid phosphatases serve many biological purposes, and proteins are designated as such based on a shared similarity in catalytic site structural arrangement (35, 36). Typically, these enzymes coordinate an Fe(III) and a divalent metal, such as Mn(II), Zn(II), or Fe(II), as part of their active site. Each metal is coordinated to 3 amino acids and shares a linking aspartic acid bridge between them, with the Fe(III) typically being mated to a histidine, an asparagine, and a tyrosine and the divalent metal being bound by two histidines and an asparagine (35, 36). Based on homology alignment of its phosphatase domain, GRA44 contains a majority of the conserved residues common to acid phosphatases (see Fig. S3 in the supplemental material). In GRA44, the aspartic acid conjugating the Fe(III) is switched to an asparagine and the asparagine conjugating the second metal is replaced by a glutamic acid. This would represent a swap in charged amino acids and should still maintain a stable charge equilibrium within the active site. The only coordinating residue unaccounted for is the tyrosine binding Fe(III) (Fig. S3). The active site of GRA44 could then be hypothesized to consist of an Asp bridge between the first metal, M(II), bound by a glutamic acid and two histidines, and the second metal, M(III), bound by an asparagine, a histidine, and an unknown seventh residue.

10.1128/mSphere.00877-19.2 FIG S2 c-Myc activation by parasites of the TATi-GRA44(HA) and complemented strains grown with or without ATc. Host cells were monitored for nuclear c-Myc staining. Results from three independent experiments are shown. The data were analyzed by analysis of variance. ****, *P* < 0.0001; ***, *P* = 0.0002; **, *P* = 0003; *, *P* = 0.01. Download FIG S2, PDF file, 0.1 MB. Copyright © 2020 Blakely et al. 2020 Blakely et al. This content is distributed under the terms of the Creative Commons Attribution 4.0 International license.

10.1128/mSphere.00877-19.3 FIG S3 Alignment of the putative phosphatase domain (PD) of GRA44 (amino acids 350 to 700) with soybean purple acid phosphatase (PAP; GenBank accession number NP_001235547), the human tartrate-resistant acid phosphatase type 5 (ACP5; GenBank accession number NP_001602), Arabidopsis thaliana purple acid phosphatase 8 (PAP8; GenBank accession number NP_001325278), and tartrate-resistant acid phosphatase type 5 (ACP5) from the wild boar (Sus scrofa) (GenBank accession number NP_999374). Amino acids known to coordinate metal in the pig ACP5 (35) are marked. *, amino acids that mate to iron; #, amino acids that bind the divalent metal in the second metal binding centers; =, the aspartic acid bridge between the two pockets. Alignment was performed with the CLUSTAL program using the DNAStar suite. Download FIG S3, PDF file, 0.05 MB. Copyright © 2020 Blakely et al. 2020 Blakely et al. This content is distributed under the terms of the Creative Commons Attribution 4.0 International license.

Acid phosphatases commonly scavenge, recycle, and transport inorganic phosphorus and have been implicated in various biological functions, such as the downregulation of prostate cell growth signaling and osteoclast bone resorption activity (37, 38). Acid phosphatases have also been implicated in phosphate acquisition from organophosphate compounds and the dephosphorylative regulation of enzymes in plants (39, 40). The GRA44 function, at least in part, is likely related to its interactions with the MYR translocon in the PV. Two plausible functions for GRA44 could be regulation of MYR component proteins by dephosphorylation or the dephosphorylation of effectors for trafficking across the PVM structure. Typically, proteins must be dephosphorylated to cross a lipid bilayer membrane, such as the PVM, and notably, both GRA16 and GRA24 have been shown to be phosphorylated, as identified by *Toxoplasma* phosphoproteome analysis (41). MYR3 also exists in a partial phosphorylated state and could be a substrate for a phosphatase, such as GRA44 (27). Consistent with the idea of phosphoregulation of the export system, the secreted kinase ROP17 has been shown to be critical for efficient effector translocation (42).

Besides the defect in c-Myc activation, the genetic disruption of GRA44 results in a propagation defect. This result is congruent with published work describing the effect of complete GRA44 knockout (13) and with the fitness score of −3.28 assigned to GRA44 through a genome-wide CRISPR screen (18). This is particularly interesting, as the disruption of other translocon members does not affect fitness to the level seen with the disruption of GRA44. For example, the relative fitness scores for MYR1, MYR2, and MYR3 are 0.88, 2.39, and 2.83, respectively, although disruption of any of these interferes with effector translocation and c-Myc activation. Similarly, disruption of the effectors GRA16, GRA24, and TgIST, which depend on MYR1 for translocation, have positive fitness scores of 1.44, 2.28, and 2.86, respectively. Thus, it is unlikely that the propagation defect exhibited by parasites lacking GRA44 is due to defects on effector translocation. Alternatively, GRA44 might play several independent roles, including nutrient acquisition, which would be consistent with the known functions of acid phosphatases.

In conclusion, we have shown GRA44 to be a secreted protein critical for *Toxoplasma* survival and propagation that plays a significant role in host manipulation and that interacts with the translocon protein MYR1. As part of its secretion to the PV, it is cleaved at an internal TEXEL site, forming two stable and colocalizing proteins. The mechanistic action by which GRA44 is involved with protein secretion to host cells remains unknown; however, due to its putative acid phosphatase domain, involvement with the dephosphorylation of trafficked proteins or members of the translocon complex is plausible. Future work on the activity and substrates of GRA44 will shed light on the regulation of effector translocation, a process central to the interactions between *Toxoplasma* and its host.

## MATERIALS AND METHODS

### Parasite and host cell culture.

All parasite lines were maintained by continuous passage through human foreskin fibroblasts (HFFs), purchased from ATCC. Parasites and HFFs were grown in Dulbecco’s modified Eagle medium (DMEM) supplemented with 10% fetal bovine serum (FBS), 2 mM/liter glutamine, 100 units penicillin/ml, and 100 μg streptomycin/ml. When pyrimethamine was included in the medium for selection, dialyzed FBS was used. All parasite and HFF cultures were grown in a humidified incubator at 37°C with 5% CO_2_. The initial parental parasite lines used were the RH strain lacking the hypoxanthine-xanthine-guanine phosphoribosyltransferase (HPT) gene, referred to as RHΔ*hpt* (43), and the RH strain lacking HPT and Ku80, referred to as RHΔ*ku80* (44, 45). For drug treatment and selection, stocks of pyrimethamine and chloramphenicol were prepared in ethanol and stocks of anhydrotetracycline (ATc) were prepared in dimethyl sulfoxide. All drugs were purchased from Sigma.

### Endogenous epitope tagging.

For C-terminal endogenous tagging of TGGT1_228170, the 3′ region directly upstream of the stop codon was amplified from RHΔ*ku80* parasite genomic DNA by PCR and inserted into the pLIC-3×HA-DHFR (45) vector at the PacI restriction site by ligation-independent cloning (LIC), facilitated by use of an InFusion HD Cloning Plus system (Clontech). The sequences of the primers for this and all reactions used in this work are presented in Table S3 in the supplemental material. Fifty micrograms of XcmI-linearized vector was transfected into RHΔ*ku80* parasites, and the resultant population was selected for the presence of the pyrimethamine-resistant dihydrofolate reductase (DHFR) allele, which is included in the vector (46). Independent clones were established by limiting dilution of the transfected population and confirmed by immunofluorescence assay and Western blotting.

10.1128/mSphere.00877-19.6 TABLE S3 Primers used in this study. Download Table S3, PDF file, 0.1 MB. Copyright © 2020 Blakely et al. 2020 Blakely et al. This content is distributed under the terms of the Creative Commons Attribution 4.0 International license.

### Exogenous gene insertion and parasite line generation.

To introduce an exogenous copy of TGGT1_228170 into parasites, we first generated a vector containing a section of the genomic TGGT1_228170 locus beginning from the start codon and going to the stop codon that included introns and a C-terminal HA epitope. The section of TGGT1_228170 in the vector was flanked by the *Toxoplasma* tubulin promoter and 5′ untranslated region (UTR) and the tubulin 3′ UTR. This was achieved by cloning a PCR amplicon of the TGGT1_228170 genomic DNA (the primers are provided in Table S3) into the NcoI and PacI sites of pTNRluc-Tub-HPT (47) using the InFusion HD Cloning Plus system for LIC. Fifty micrograms of the resulting vector, pTub-Gra44-HPT, linearized with ScaI, was transfected into RHΔ*hpt* parasites. The transfected population was selected for HPT by adding mycophenolic acid (50 μg/ml) and xanthine (50 μg/ml) to the medium. Independent clones were established by limiting dilution. For mutant variations of the exogenously expressed TGGT1_228170, TEXEL deletions, a MYC epitope tag insertion, and TEXEL2 point mutations were introduced into pTub-Gra44-HPT using a Q5 site-directed mutagenesis kit (New England Biolabs [NEB]), and TEXEL point mutations were accomplished similarly with a QuikChange site-directed mutagenesis kit (Agilent).

### Development of GRA44 conditional knockdown parasite line.

To generate the GRA44 conditional knockout strain, we introduced a cassette encoding a drug-selective marker, a transactivator (TATi) protein, and a Tet response element (TRE) just upstream of the GRA44 start codon. This TATi cassette was amplified from the vector pT8TATi-Gra44-HX-tetO7S (48) with primers that include areas of homology upstream of GRA44 to facilitate homologous recombination. One microgram of this PCR amplicon was transfected into the RHΔ*ku80* parasites that expressed endogenously HA-tagged GRA44. To drive the insertion of the TATi cassette, we cotransfected the PCR amplicon with 2 μg of a vector expressing Cas9 and a guide RNA targeting the TGGT1_228170 locus upstream of the start codon. This vector was made using pSAG1-Cas9-GFP-pU6-sgUPRT (49) as a template, and the sequences encoding the guide RNA were introduced with the Q5 site-directed mutagenesis kit (NEB). Parasites transfected with the TATi cassette and Cas9 vector were selected for HPT, and independent clones were established by limiting dilution. Correct integration of the TATi insert cassette was validated by PCR. The resulting strain was designated TATi-GRA44(HA).

### Complementation of conditional knockdown line.

To complement the knockdown strain, a wild-type copy of TGGT1_228170 that was driven by a *Toxoplasma* tubulin promoter and that included a C-terminal MYC epitope tag was targeted to the inactive *ku80* locus of the TATi-GRA44(HA) strain using CRISPR/Cas9 to assist with integration. The insertional cassette, which includes the tubulin-driven TGGT1_228170 and a chloramphenicol resistance gene (50), was amplified by PCR from plasmid pTub-Gra44-myc-CmR with primers that included homology segments to the *ku80* locus (Table S3).The pTub-Gra44-myc-CmR vector was constructed with InFusion HD Cloning Plus system-assisted LIC cloning by inserting the GRA44 gene with an appended C-terminal MYC tag, amplified from pTub-Gra44-HPT, into the pLIC-SMGFP-CmR vector backbone (51), replacing the soluble modified green fluorescent protein (SMGFP) tag and upstream region. One microgram of this PCR amplicon was cotransfected with 2 μg of the Cas9 vector encoding a small guide RNA targeting the *ku80* locus.

### SDS-PAGE and Western blot analysis.

For detection of protein in lysates from extracellular parasite samples, parasites were allowed to undergo natural egress and were then collected, centrifuged, and washed 2 times with cold phosphate-buffered saline (PBS) (10 min, 1,000 × *g*). For analysis of intracellular parasite protein lysates, host cell monolayers were washed 2 times with cold PBS, scraped, and centrifuged for 10 min at 1,000 × *g*. Parasite samples were resuspended in 2× sample loading buffer with 5% β-mercaptoethanol and boiled for 5 min at 98°C. The boiled samples were frozen at −20°C and then thawed and reboiled for 5 min at 98°C before gel loading. SDS-PAGE and Western blotting were performed by standard methods, as previously described (52).

For Western blot analysis of GRA44 conditional mutant strains, parasites were first grown under normal conditions for 24 h and then syringe lysed with a 27-gauge needle. Fresh host cells were infected with an equal quantity of syringe-lysed parasites and grown for 24 or 48 h with or without 1 μg/ml ATc. For analysis of protein lysates from extracellular parasite samples, host cells were scraped, and parasites were released by passing them through a syringe and centrifuged for 10 min at 1,000 × *g*. For analysis of intracellular parasite protein lysates, host cell monolayers were washed with cold PBS, scraped, and centrifuged for 10 min at 1,000 × *g*. The resulting samples were resuspended in 200 μl radioimmunoprecipitation assay lysis buffer (50 mM Tris, 150 mM NaCl, 0.1% SDS, 0.5% sodium deoxycholate, 1% Triton X-100) including a protease/phosphatase inhibitor cocktail (Cell Signaling Technology) and incubated on ice for 1 h, sonicated 2 times for 15 s each time with 1-min rests on ice, and centrifuged (20,000 × *g*, 15 min, 4°C). The supernatants were combined with 4× SDS loading buffer with 10% β-mercaptoethanol and boiled for 5 min at 98°C. The boiled SDS samples were frozen at −20°C and then thawed and reboiled for 5 min at 98°C before gel loading. SDS-PAGE and Western blotting were performed by standard methods as described above. Uncropped original images for all Western blots are included as Data Set S2.

10.1128/mSphere.00877-19.8 DATA SET S2 Original images of the Western blots shown in the figures. Download Data Set S2, PDF file, 8.7 MB. Copyright © 2020 Blakely et al. 2020 Blakely et al. This content is distributed under the terms of the Creative Commons Attribution 4.0 International license.

The primary antibodies used for Western blotting included rabbit anti-HA at a dilution of 1:1,000 (Cell Signaling Technologies), rabbit anti-MYC at a dilution of 1:1,000 (Cell Signaling Technologies), mouse anti-SAG1 at a dilution of 1:2,000 (Genway), and mouse anti-MYR1 antibodies at 1:1,000 (26, 27). The secondary antibodies used included peroxidase-conjugated goat anti‐mouse and anti-rabbit immunoglobulins and were used at a 1:10,000 dilution.

### IFAs.

For all immunofluorescence assays (IFA), HFFs were grown to confluence on 1.5-mm glass coverslips and infected with parasites, which were allowed to grow for 20 h prior to fixation with 4% paraformaldehyde for 20 min. Cells were washed once with PBS after fixation and then permeabilized and blocked with a solution of 3% bovine serum albumin (BSA)–0.2% Triton X-100 in PBS for 15 to 20 min. Coverslips were incubated with primary antibodies in 3% BSA–0.2% Triton X-100 in PBS for 1 h at room temperature and washed five times with PBS. Finally, the cultures were incubated with fluorophore-conjugated secondary antibodies for 1 h at room temperature in 3% BSA in PBS and then washed five times with PBS and mounted on glass slides with Vectashield mounting medium containing DAPI (Vector Laboratories). The primary antibodies used were rabbit anti-HA at 1:1,000, mouse anti-MYC at 1:1,000 (Cell Signaling Technology), rat anti-HA at 1:2,000 (Roche), rabbit anti-human c-Myc at 1:1,000 (Abcam), mouse anti-gra5 (Biotem) at 1:1,000, and mouse anti-gra7 at 1:1,000. The secondary antibodies used (Life Technologies) were Alexa Fluor 488-conjugated goat anti-rabbit or goat anti-rat immunoglobulin, Alexa Fluor 594-conjugated goat anti-mouse or goat anti-rabbit immunoglobulin, and Alexa Fluor 647-conjugated goat anti-mouse immunoglobulin. All secondary antibodies were used at 1:2,000. Images were taken on a Nikon Eclipse 80i microscope using a Nikon DS-Qi1Mc camera and NIS Elements AR (v3.0) software.

### Immunoprecipitation and co-IP experiments.

Infected host cells were washed 2 times with cold PBS and scraped from the flask surface to collect intracellular parasites, which were centrifuged for 10 min at 1,000 × *g* and resuspended in 200 μl ice-cold IP lysis buffer (Pierce, Thermo Scientific) containing protease and phosphatase inhibitors (Cell Signaling Technology). The lysate was incubated on ice for 1 h, sonicated on ice 2 times for 15 s each time, and centrifuged for 15 min at 20,000 × *g* and 4°C. The supernatant was collected and incubated with magnetic beads conjugated to either mouse IgG or primary antibody (Pierce, Thermo Scientific) for 1 h at 4°C with rocking. The incubated beads were separated from the solution with a magnet and washed with IP lysis buffer (Pierce, Thermo Scientific) plus inhibitors 3 times and either stored in 8 M urea at −80°C for downstream mass spectrometric analysis or directly eluted into 2× SDS sample loading buffer–5% β-mercaptoethanol, boiled for 5 min at 98°C, and stored at −20°C for Western blot analysis. SDS-PAGE and Western blotting were performed as outlined above. Protein analysis by mass spectrometry was completed by the Indiana University School of Medicine Proteomics Core facility as previously described (52).

### Plaque assays.

Twelve-well plates were infected with 500 parasites/well of freshly syringe-lysed parasites, and the parasites were grown undisturbed for 5 days before fixation with methanol for 5 min. The wells were stained with crystal violet, and the plaque images were quantified with ImageJ software using the ColonyArea plug-in (52).

### HFF c-Myc response assay and quantitation.

The parasites used for c-Myc assays were grown for 48 h with or without 1 μg/ml ATc and syringe lysed prior to infection of host cell coverslips. Coverslips of confluent HFF monolayers pretreated for 24 h with FBS-free medium were infected and fixed at 19 h postinfection. IFAs for human c-Myc, HA, and DAPI were performed as described above. Images of phase-contrast, DAPI, HA, MYC, and c-Myc channels were acquired for at least 20 vacuoles under each experimental condition and exported to ImageJ software. Infected host cell nuclei were identified from merged-channel images and quantitated for c-Myc expression from images of the c-Myc channel alone. Measurements of the mean pixel intensity within host nucleus boundaries of singly infected cells containing PVs with greater than one parasite were taken. Measurements from triplicate experiments were averaged.

## References

[1] Jones JL, Parise ME, Fiore AE 2014 Neglected parasitic infections in the United States: Toxoplasmosis. Am J Trop Med Hyg 90:794–799. doi:10.4269/ajtmh.13-0722. 24808246 PMC4015566

[2] Israelski D, Remington J 1993 Toxoplasmosis in patients with cancer. Clin Infect Dis 17(Suppl 2):S423–S435. doi:10.1093/clinids/17.Supplement_2.S423. 8274608

[3] Luft B, Remington J 1992 Toxoplasmic encephalitis in AIDS. Clin Infect Dis 15:211–222. doi:10.1093/clinids/15.2.211. 1520757

[4] Slavin MA, Meyers JD, Remington JS, Hackman RC 1994 *Toxoplasma gondii* infection in marrow transplant recipients: a 20-year experience. Bone Marrow Transplantation 13:549–557. 8054907

[5] Wong S, Remington J 1994 Toxoplasmosis in pregnancy. Clin Infect Dis 18:853–861. doi:10.1093/clinids/18.6.853. 8086543

[6] Hakimi M-A, Olias P, Sibley LD 2017 *Toxoplasma* effectors targeting host signaling and transcription. Clin Microbiol Rev 30:615–645. doi:10.1128/CMR.00005-17. 28404792 PMC5475222

[7] Blader IJ, Saeij JP 2009 Communication between *Toxoplasma gondii* and its host: impact on parasite growth, development, immune evasion, and virulence. APMIS 117:458–476. doi:10.1111/j.1600-0463.2009.02453.x. 19400868 PMC2810527

[8] Naor A, Panas MW, Marino N, Coffey MJ, Tonkin CJ, Boothroyd JC 2018 MYR1-dependent effectors are the major drivers of a host cell’s early response to *Toxoplasma*, including counteracting MYR1-independent effects. mBio 9:e02401-17. doi:10.1128/mBio.02401-17. 29615509 PMC5885026

[9] Saeij JPJ, Coller S, Boyle JP, Jerome ME, White MW, Boothroyd JC 2007 *Toxoplasma* co-opts host gene expression by injection of a polymorphic kinase homologue. Nature 445:324–327. doi:10.1038/nature05395. 17183270 PMC2637441

[10] Taylor S, Barragan A, Su C, Fux B, Fentress SJ, Tang K, Beatty WL, Hajj HE, Jerome M, Behnke MS, White M, Wootton JC, Sibley LD 2006 A secreted serine-threonine kinase determines virulence in the eukaryotic pathogen *Toxoplasma gondii*. Science 314:1776–1780. doi:10.1126/science.1133643. 17170305

[11] Ong YC, Reese ML, Boothroyd JC 2010 *Toxoplasma* rhoptry protein 16 (ROP16) subverts host function by direct tyrosine phosphorylation of STAT6. J Biol Chem 285:28731–28740. doi:10.1074/jbc.M110.112359. 20624917 PMC2937901

[12] Hermanns T, Müller UB, Könen-Waisman S, Howard JC, Steinfeldt T 2016 The *Toxoplasma gondii* rhoptry protein ROP18 is an Irga6-specific kinase and regulated by the dense granule protein GRA7. Cell Microbiol 18:244–259. doi:10.1111/cmi.12499. 26247512 PMC5061101

[13] Coffey MJ, Dagley LF, Seizova S, Kapp EA, Infusini G, Roos DS, Boddey JA, Webb AI, Tonkin CJ 2018 Aspartyl protease 5 matures dense granule proteins that reside at the host-parasite interface in *Toxoplasma gondii*. mBio 9:e01796-18. doi:10.1128/mBio.01796-18. 30377279 PMC6212819

[14] Gao XJ, Feng JX, Zhu S, Liu XH, Tardieux I, Liu LX 2014 Protein phosphatase 2C of *Toxoplasma gondii* interacts with human SSRP1 and negatively regulates cell apoptosis. Biomed Environ Sci 27:883–893. doi:10.3967/bes2014.130. 25374021

[15] Gilbert LA, Ravindran S, Turetzky JM, Boothroyd JC, Bradley PJ 2007 *Toxoplasma gondii* targets a protein phosphatase 2C to the nuclei of infected host cells. Eukaryot Cell 6:73–83. doi:10.1128/EC.00309-06. 17085638 PMC1800361

[16] Mann T, Beckers C 2001 Characterization of the subpellicular network, a filamentous membrane skeletal component in the parasite *Toxoplasma gondii*. Mol Biochem Parasitol 115:257–268. doi:10.1016/s0166-6851(01)00289-4. 11420112

[17] Tonkin ML, Beck JR, Bradley PJ, Boulanger MJ 2014 The inner membrane complex sub-compartment proteins critical for replication of the apicomplexan parasite *Toxoplasma gondii* adopt a pleckstrin homology fold. J Biol Chem 289:13962–13973. doi:10.1074/jbc.M114.548891. 24675080 PMC4022867

[18] Sidik SM, Huet D, Ganesan SM, Huynh M-H, Wang T, Nasamu AS, Thiru P, Saeij JPJ, Carruthers VB, Niles JC, Lourido S 2016 A genome-wide CRISPR screen in *Toxoplasma* identifies essential apicomplexan genes. Cell 166:1423–1435.e12. doi:10.1016/j.cell.2016.08.019. 27594426 PMC5017925

[19] Khosh-Naucke M, Becker J, Mesén-Ramírez P, Kiani P, Birnbaum J, Fröhlke U, Jonscher E, Schlüter H, Spielmann T 2017 Identification of novel parasitophorous vacuole proteins in P falciparum parasites using BioID. Int J Med Microbiol 308:13–24. doi:10.1016/j.ijmm.2017.07.007. 28784333

[20] Nadipuram SM, Kim EW, Vashisht AA, Lin AH, Bell HN, Coppens I, Wohlschlegel JA, Bradley PJ 2016 In vivo biotinylation of the *Toxoplasma* parasitophorous vacuole reveals novel dense granule proteins important for parasite growth and pathogenesis. mBio 7:e00808-16. doi:10.1128/mBio.00808-16. 27486190 PMC4981711

[21] Curt-Varesano A, Braun L, Ranquet C, Hakimi M-A, Bougdour A 2016 The aspartyl protease TgASP5 mediates the export of the *Toxoplasma* GRA16 and GRA24 effectors into host cells. Cell Microbiol 18:151–167. doi:10.1111/cmi.12498. 26270241

[22] Coffey MJ, Sleebs BE, Uboldi AD, Garnham A, Franco M, Marino ND, Panas MW, Ferguson DJ, Enciso M, O'Neill MT, Lopaticki S, Stewart RJ, Dewson G, Smyth GK, Smith BJ, Masters SL, Boothroyd JC, Boddey JA, Tonkin CJ 2015 An aspartyl protease defines a novel pathway for export of *Toxoplasma* proteins into the host cell. Elife 4:e10809. doi:10.7554/eLife.10809. 26576949 PMC4764566

[23] Meissner M, Brecht S, Bujard H, Soldati D 2001 Modulation of myosin A expression by a newly established tetracycline repressor-based inducible system in *Toxoplasma gondii*. Nucleic Acids Res 29:E115. doi:10.1093/nar/29.22.e115. 11713335 PMC92585

[24] Sheiner L, Demerly JL, Poulsen N, Beatty WL, Lucas O, Behnke MS, White MW, Striepen B 2011 A systematic screen to discover and analyze apicoplast proteins identifies a conserved and essential protein import factor. PLoS Pathog 7:e1002392. doi:10.1371/journal.ppat.1002392. 22144892 PMC3228799

[25] Teo G, Liu G, Zhang J, Nesvizhskii AI, Gingras A-C, Choi H 2014 SAINTexpress: improvements and additional features in Significance Analysis of INTeractome software. J Proteomics 100:37–43. doi:10.1016/j.jprot.2013.10.023. 24513533 PMC4102138

[26] Franco M, Panas MW, Marino ND, Lee M-C, Buchholz KR, Kelly FD, Bednarski JJ, Sleckman BP, Pourmand N, Boothroyd JC 2016 A novel secreted protein, MYR1, is central to Toxoplasma’s manipulation of host cells. mBio 7:e02231-15. doi:10.1128/mBio.02231-15. 26838724 PMC4742717

[27] Marino ND, Panas MW, Franco M, Theisen TC, Naor A, Rastogi S, Buchholz KR, Lorenzi HA, Boothroyd JC 2018 Identification of a novel protein complex essential for effector translocation across the parasitophorous vacuole membrane of *Toxoplasma gondii*. PLoS Pathog 14:e1006828. doi:10.1371/journal.ppat.1006828. 29357375 PMC5794187

[28] Reese ML, Shah N, Boothroyd JC 2014 The *Toxoplasma* pseudokinase ROP5 is an allosteric inhibitor of the immunity-related GTPases. J Biol Chem 289:27849–27858. doi:10.1074/jbc.M114.567057. 25118287 PMC4183819

[29] Delorme-Walker V, Abrivard M, Lagal V, Anderson K, Perazzi A, Gonzalez V, Page C, Chauvet J, Ochoa W, Volkmann N, Hanein D, Tardieux I 2012 Toxofilin upregulates the host cortical actin cytoskeleton dynamics, facilitating *Toxoplasma* invasion. J Cell Sci 125:4333–4342. doi:10.1242/jcs.103648. 22641695 PMC3516439

[30] Guo H, Gao Y, Jia H, Moumouni PFA, Masatani T, Liu M, Lee S-H, Galon EM, Li J, Li Y, Tumwebaze MA, Benedicto B, Xuan X 2019 Characterization of strain-specific phenotypes associated with knockout of dense granule protein 9 in *Toxoplasma gond*ii. Mol Biochem Parasitol 229:53–61. doi:10.1016/j.molbiopara.2019.01.003. 30849416

[31] Bougdour A, Durandau E, Brenier-Pinchart M-P, Ortet P, Barakat M, Kieffer S, Curt-Varesano A, Curt-Bertini R-L, Bastien O, Coute Y, Pelloux H, Hakimi M-A 2013 Host cell subversion by *Toxoplasma* GRA16, an exported dense granule protein that targets the host cell nucleus and alters gene expression. Cell Host Microbe 13:489–500. doi:10.1016/j.chom.2013.03.002. 23601110

[32] Shastri AJ, Marino ND, Franco M, Lodoen MB, Boothroyd JC 2014 GRA25 is a novel virulence factor of *Toxoplasma gondii* and influences the host immune response. Infect Immun 82:2595–2605. doi:10.1128/IAI.01339-13. 24711568 PMC4019154

[33] Pernas L, Adomako-Ankomah Y, Shastri AJ, Ewald SE, Treeck M, Boyle JP, Boothroyd JC 2014 *Toxoplasma* effector MAF1 mediates recruitment of host mitochondria and impacts the host response. PLoS Biol 12:e1001845. doi:10.1371/journal.pbio.1001845. 24781109 PMC4004538

[34] Cygan AM, Theisen TC, Mendoza AG, Marino ND, Panas MW, Boothroyd JC 2020 Coimmunoprecipitation with MYR1 identifies three additional proteins within the Toxoplasma gondii parasitophorous vacuole required for translocation of dense granule effectors into host cells. mSphere 5:e00858-19. doi:10.1128/mSphere.00858-19. 32075880 PMC7031616

[35] Guddat LW, McAlpine AS, Hume D, Hamilton S, de Jersey J, Martin JL 1999 Crystal structure of mammalian purple acid phosphatase. Structure 7:757–767. doi:10.1016/s0969-2126(99)80100-2. 10425678

[36] Klabunde T, Krebse B 1997 The dimetal center in purple acid phosphatases, p 177–198. In Hill HAO, Sadler PJ, Thomson AJ (ed), Metal sites in proteins and models: phosphatases, Lewis acids and vanadium. Springer, Berlin, Germany.

[37] Meng TC, Lin MF 1998 Tyrosine phosphorylation of c-ErbB-2 is regulated by the cellular form of prostatic acid phosphatase in human prostate cancer cells. J Biol Chem 273:22096–22104. doi:10.1074/jbc.273.34.22096. 9705354

[38] Clark SA, Ambrose WW, Anderson TR, Terrell RS, Toverud SU 1989 Ultrastructural localization of tartrate-resistant, purple acid phosphatase in rat osteoclasts by histochemistry and immunocytochemistry. J Bone Miner Res 4:399–405. doi:10.1002/jbmr.5650040315. 2763875

[39] Bozzo GG, Raghothama KG, Plaxton WC 2002 Purification and characterization of two secreted purple acid phosphatase isozymes from phosphate-starved tomato (*Lycopersicon esculentum*) cell cultures. Eur J Biochem 269:6278–6286. doi:10.1046/j.1432-1033.2002.03347.x. 12473124

[40] Kaida R, Serada S, Norioka N, Norioka S, Neumetzler L, Pauly M, Sampedro J, Zarra I, Hayashi T, Kaneko TS 2010 Potential role for purple acid phosphatase in the dephosphorylation of wall proteins in tobacco cells. Plant Physiol 153:603–610. doi:10.1104/pp.110.154138. 20357138 PMC2879787

[41] Treeck M, Sanders JL, Elias JE, Boothroyd JC 2011 The phosphoproteomes of *Plasmodium falciparum* and *Toxoplasma gondii* reveal unusual adaptations within and beyond the parasites’ boundaries. Cell Host Microbe 10:410–419. doi:10.1016/j.chom.2011.09.004. 22018241 PMC3254672

[42] Panas MW, Ferrel A, Naor A, Tenborg E, Lorenzi HA, Boothroyd JC 2019 Translocation of dense granule effectors across the parasitophorous vacuole membrane in *Toxoplasma*-infected cells requires the activity of ROP17, a rhoptry protein kinase. mSphere 4:e00276-19. doi:10.1128/mSphere.00276-19. 31366709 PMC6669336

[43] Donald RG, Carter D, Ullman B, Roos DS 1996 Insertional tagging, cloning, and expression of the *Toxoplasma gondii* hypoxanthine-xanthine-guanine phosphoribosyltransferase gene. Use as a selectable marker for stable transformation. J Biol Chem 271:14010–14019. doi:10.1074/jbc.271.24.14010. 8662859

[44] Fox B, Ristuccia J, Gigley J, Bzik D 2009 Efficient gene replacements in *Toxoplasma gondii* strains deficient for nonhomologous end joining. Eukaryot Cell 8:520–529. doi:10.1128/EC.00357-08. 19218423 PMC2669201

[45] Huynh M, Carruthers V 2009 Tagging of endogenous genes in a *Toxoplasma gondii* strain lacking Ku80. Eukaryot Cell 8:530–539. doi:10.1128/EC.00358-08. 19218426 PMC2669203

[46] Donald RG, Roos DS 1993 Stable molecular transformation of *Toxoplasma gondii*: a selectable dihydrofolate reductase-thymidylate synthase marker based on drug-resistance mutations in malaria. Proc Natl Acad Sci U S A 90:11703–11707. doi:10.1073/pnas.90.24.11703. 8265612 PMC48052

[47] Saeij JPJ, Arrizabalaga G, Boothroyd JC 2008 A cluster of four surface antigen genes specifically expressed in bradyzoites, SAG2CDXY, plays an important role in *Toxoplasma gondii* persistence. Infect Immun 76:2402–2410. doi:10.1128/IAI.01494-07. 18347037 PMC2423105

[48] Salamun J, Kallio JP, Daher W, Soldati-Favre D, Kursula I 2014 Structure of *Toxoplasma gondii* coronin, an actin-binding protein that relocalizes to the posterior pole of invasive parasites and contributes to invasion and egress. FASEB J 28:4729–4747. doi:10.1096/fj.14-252569. 25114175

[49] Shen B, Brown KM, Lee TD, Sibley LD 2014 Efficient gene disruption in diverse strains of *Toxoplasma gondii* using CRISPR/CAS9. mBio 5:e01114-14. doi:10.1128/mBio.01114-14. 24825012 PMC4030483

[50] Kim K, Soldati D, Boothroyd J 1993 Gene replacement in *Toxoplasma gondii* with chloramphenicol acetyltransferase as selectable marker. Science 262:911–914. doi:10.1126/science.8235614. 8235614

[51] Hortua Triana MA, Márquez-Nogueras KM, Chang L, Stasic AJ, Li C, Spiegel KA, Sharma A, Li Z-H, Moreno S 2018 Tagging of weakly expressed *Toxoplasma gondii* calcium-related genes with high-affinity tags. J Eukaryot Microbiol 65:709–721. doi:10.1111/jeu.12626. 29672999 PMC6175649

[52] Yang C, Broncel M, Dominicus C, Sampson E, Blakely WJ, Treeck M, Arrizabalaga G 2019 A plasma membrane localized protein phosphatase in *Toxoplasma gondii*, PPM5C, regulates attachment to host cells. Sci Rep 9:5924. doi:10.1038/s41598-019-42441-1. 30976120 PMC6459975

