# Supplementary material for: The Secreted Acid Phosphatase Domain-Containing GRA44 from Toxoplasma gondii Is Required for c-Myc Induction in Infected Cells
Source: mSphere. 2020 Feb 19;5(1):e00877-19. doi: 10.1128/mSphere.00877-19 (PMC7031617; doi:10.1128/mSphere.00877-19)
Supplement: TABLE S1 [file mSphere.00877-19-st001.pdf]

ID number	Product Description	Fitness score
TGGT1_283720	phosphotyrosyl phosphate activator (ptpa) protein	-4.71
TGGT1_224920	hypothetical protein	-4.56
TGGT1_311290	protein tyrosine phosphatase family protein, ptpla protein	-3.86
TGGT1_283590A	NLI interacting factor family phosphatase	-3.76
TGGT1_219320	acid phosphatase GAP50	-3.74
TGGT1_228170	inner membrane complex protein IMC2A	-3.28
TGGT1_276210	phosphoglycerate mutase family protein	-2.94
TGGT1_216600	exonuclease III APE	-2.67
TGGT1_237410	protein phosphatase 2C domain-containing protein	-2.01
TGGT1_204080	histidine acid phosphatase superfamily protein	-1.99
TGGT1_243990	Dullard family phosphatase domain-containing protein	-1.94
TGGT1_224220	serine/threonine-protein phosphatase PP2A catalytic subunit	-1.5
TGGT1_305910	hypothetical protein	-1.31
TGGT1_252380	hypothetical protein	-1.01
TGGT1_259960	Nucleoside-diphosphatase	-0.67
TGGT1_204410	endonuclease/exonuclease/phosphatase family protein	-0.49
TGGT1_201630A	protein phosphatase 2C domain-containing protein	-0.06
TGGT1_277720	GDA1/CD39 (nucleoside phosphatase) family protein	0.28
TGGT1_244450	protein phosphatase 2C domain-containing protein	0.51
TGGT1_228160	acid phosphatase	0.55
TGGT1_278878	GDA1/CD39 (nucleoside phosphatase) family protein	0.6
TGGT1_268770	dual specificity phosphatase, catalytic domain-containing protein	0.71
TGGT1_278882	GDA1/CD39 (nucleoside phosphatase) family protein	0.71
TGGT1_276920	protein phosphatase 2C domain-containing protein	0.75
TGGT1_308950	histidine acid phosphatase superfamily protein	0.77
TGGT1_225290	GDA1/CD39 (nucleoside phosphatase) family protein	0.81
TGGT1_278510	protein phosphatase 2C domain-containing protein	1.01
TGGT1_237500	protein phosphatase 2C domain-containing protein	1.13
TGGT1_270320	protein phosphatase 2C domain-containing protein	1.24
TGGT1_297650	Ser/Thr phosphatase family protein	1.52
TGGT1_222840	Ser/Thr phosphatase family protein	2.25

Table S1
