# Supplementary figures and images for: The Secreted Acid Phosphatase Domain-Containing GRA44 from Toxoplasma gondii Is Required for c-Myc Induction in Infected Cells
Source: mSphere. 2020 Feb 19;5(1):e00877-19. doi: 10.1128/mSphere.00877-19 (PMC7031617; doi:10.1128/mSphere.00877-19)

A.

|                 |   |   |   |   |     |
|-----------------|---|---|---|---|-----|
| PEXEL consensus | R | X | L | X | EDQ |
| GRA44 TEXEL 2   | R | R | L | L | E   |
| GRA44 R1348A    | A | R | L | L | E   |
| GRA44 L1350A    | R | R | A | L | E   |
| GRA44 E1352A    | R | R | L | L | A   |

B.

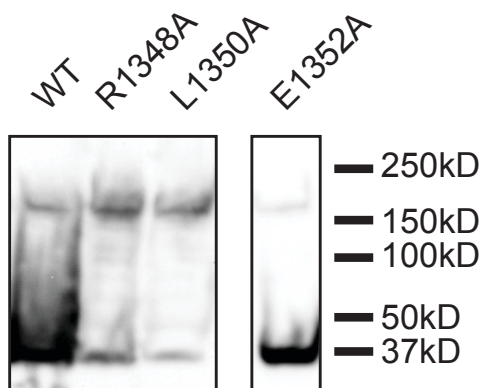

C.

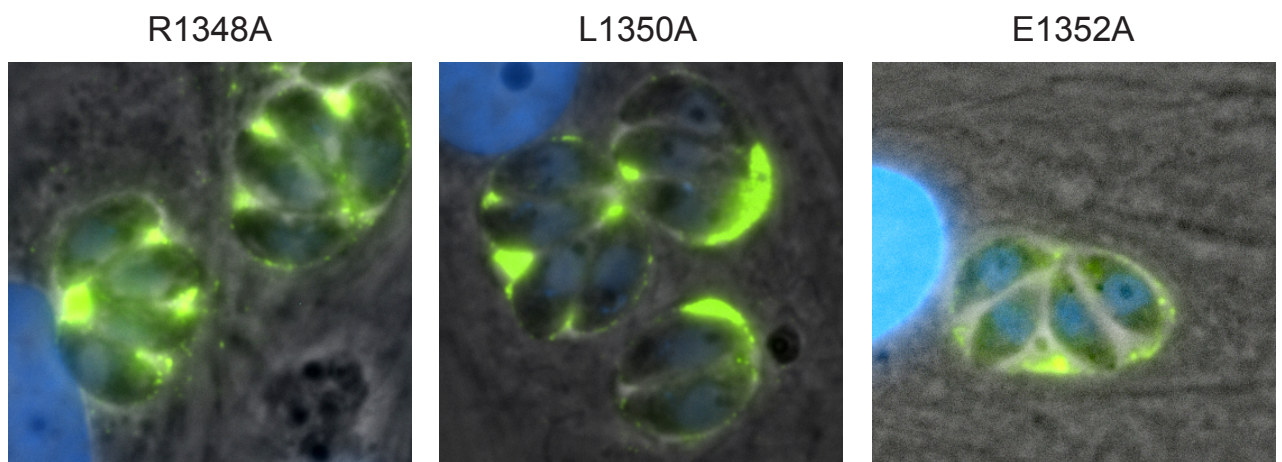

Supplement: FIG S1 [file mSphere.00877-19-sf001.pdf]

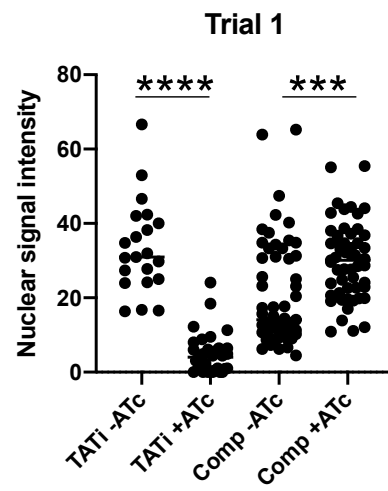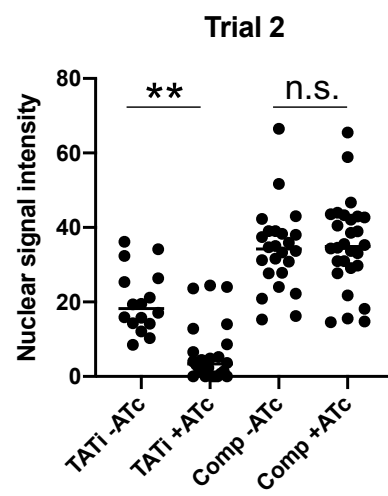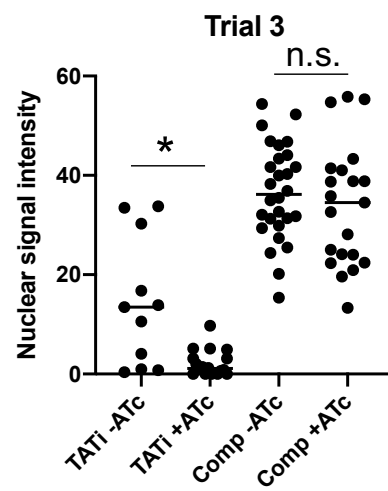

Supplement: FIG S2 [file mSphere.00877-19-sf002.pdf]
