# Supplementary material for: The Secreted Acid Phosphatase Domain-Containing GRA44 from Toxoplasma gondii Is Required for c-Myc Induction in Infected Cells
Source: mSphere. 2020 Feb 19;5(1):e00877-19. doi: 10.1128/mSphere.00877-19 (PMC7031617; doi:10.1128/mSphere.00877-19)
Supplement: TABLE S2 [file mSphere.00877-19-st002.pdf]

ID number	Product description	total peptides IPs	Total peptides controls	Fold Change	SAINT Score
TGGT1_316250	GRA45	111	0	INF	1
TGGT1_262960	putative U1 snRNP-associated protein Usp106	44	0	INF	1
TGGT1_204340	hypothetical protein	39	0	INF	1
TGGT1_254470	MYR1	30	0	INF	1
TGGT1_319340	GRA52	27	0	INF	1
TGGT1_279100	MAF1 copy	20	0	INF	1
TGGT1_309820	ribosomal protein RPL11	15	0	INF	1
TGGT1_228170	IMC2A /GRA44	1023	19	54	1
TGGT1_251540	GRA9	49	1	49	1
TGGT1_203600	GRA50	32	1	32	1
TGGT1_304955	serine/threonine specific protein phosphatase	31	1	31	1
TGGT1_207840	ribosomal protein RPS17	47	2	24	1
TGGT1_315610	hypothetical protein	21	1	21	1
TGGT1_203290	GRA34	13	1	13	1
TGGT1_266070	ribosomal protein RPL31	13	1	13	1
TGGT1_270320	protein phosphatase 2C domain-containing protein	13	0	INF	0.99
TGGT1_258870	hypothetical protein	33	2	17	0.99
TGGT1_311720	chaperonin protein BiP	140	26	5.4	0.99
TGGT1_226240	putative bud site selection protein	7	0	INF	0.97
TGGT1_216770	hypothetical protein	6	0	INF	0.96
TGGT1_242330	ribosomal protein RPS5	60	12	5	0.95
TGGT1_220950	MAF1 copy	19	2	9.5	0.94
TGGT1_270240	MAG1	112	35	3.2	0.94
TGGT1_200360	hypothetical protein	24	3	8	0.92
TGGT1_290700	GRA25	15	1	15	0.91
TGGT1_258458	hypothetical protein	10	1	10	0.89
TGGT1_262050	rhoptry kinase family protein ROP39	39	2	20	0.88
TGGT1_410360	MAF1 copy	19	2	9.5	0.87
TGGT1_248480	ribosomal protein RPS9	32	5	6.4	0.86
TGGT1_231140	ribosomal protein RPS25	14	2	7	0.85
TGGT1_247440	GRA33	15	2	7.5	0.84
TGGT1_229480	putative calcium binding protein precursor	26	4	6.5	0.84
TGGT1_208830	GRA16	14	2	7	0.83
TGGT1_410370	MAF1 copy	23	4	5.7	0.8
TGGT1_267400	ribosomal protein RPL32	26	5	5.2	0.8

Table S2
