# Supplementary material for: The Secreted Acid Phosphatase Domain-Containing GRA44 from Toxoplasma gondii Is Required for c-Myc Induction in Infected Cells
Source: mSphere. 2020 Feb 19;5(1):e00877-19. doi: 10.1128/mSphere.00877-19 (PMC7031617; doi:10.1128/mSphere.00877-19)
Supplement: DATA SET S1 [file mSphere.00877-19-sd001.pdf]

Accession number (column A).) T-test results for HA and IgG comparisons (column C). The fold change column is the HA fold change relative to the IgG sample (column D). Number of spectral counts for experimental (columns E to G) and control (columns H to J). Saint analysis (columns K to M): FC-A: Primary FC score (column K) estimates the background by averaging the spectral counts across the selected controls. FC-B: Secondary FC score (column L) estimates the background by combining the top 3 values for each prey. SP (column D) SAINT probability value of proteins. Proteins that felt within the criteria for putative GRA44 interactors are highlighted.

Accession Number	Identified Proteins (1181/1191)	T-Test (p-value): (p < 0.05)	Fold Change by Category	HA beads Total Peptides Assay 1	HA beads Total Peptides Assay 2	HA beads Total Peptides Assay 3	IgG beads Total Peptides Assay 1	IgG beads Total Peptides Assay 2	IgG beads Total Peptides Assay 3	BAIT_FC_A	BAIT_FC_B	BAIT_SP
TGGT1_200360	hypothetical protein	0.0078	8	10	7	7	3	0	0	5.47	5.42	0.92
TGGT1_201390	hypothetical protein	0.72	1.5	2	1	0	2	0	0	1.27	1.19	0.14
TGGT1_201760	hypothetical protein	0.16	0	0	0	0	2	4	0	0	0	0
TGGT1_201780	microneme protein MIC2	0.68	2	4	0	0	2	0	0	1.29	1.07	0.27
TGGT1_202200	hypothetical protein	0.68	0.5	1	0	0	2	0	0	0.85	0.83	0
TGGT1_202500	GAPM1a	0.18	0.4	4	1	1	6	8	2	0.4	0.39	0
TGGT1_202550	NLI interacting factor family phosphatase	0.52	0.5	2	0	0	2	2	0	0.62	0.57	0.02
TGGT1_203290	hypothetical protein	0.0011	13	4	5	4	1	0	0	4.6	4.42	1
TGGT1_203310	dense granule protein GRA7	0.14	2.3	21	19	9	14	4	3	2.33	2.26	0.1
TGGT1_203600	hypothetical protein	0.02	32	7	16	9	1	0	0	10.48	9.22	1
TGGT1_204020	ribosomal protein RPL8	0.37	4.4	33	4	3	9	0	0	3.72	2.93	0.33
TGGT1_204050	subtilisin SUB1	0.51	0.2	1	0	0	4	0	0	0.65	0.64	0
TGGT1_204310	hypothetical protein	0.37	INF	3	0	0	0	0	0	1.63	1.43	0.33
TGGT1_204340	hypothetical protein	0.0029	INF	15	15	9	0	0	0	14.03	13.66	1
TGGT1_205250	rhoptry protein ROP18	0.45	2	23	5	4	8	4	4	1.5	1.37	0.33
TGGT1_205340	ribosomal protein RPS12	0.85	0.8	3	0	0	4	0	0	0.88	0.77	0.1
TGGT1_205470	putative translation elongation factor 2 family protein	0.77	1.6	11	0	0	7	0	0	1.33	0.8	0.28
TGGT1_205700	cyclophilin precursor	0.85	0.8	3	0	0	4	0	0	0.88	0.77	0.1
TGGT1_206690	glideosome-associated protein with multiple-membrane spans GAPM2B	0.23	0.2	1	0	1	7	2	1	0.43	0.41	0
TGGT1_207440	ribosomal protein RPS4	0.22	2.3	17	10	5	9	3	2	2.05	2.01	0.28
TGGT1_207840	ribosomal protein RPS17	0.0068	24	21	15	11	1	0	1	9.52	9.47	1
TGGT1_207960	hypothetical protein	0.16	0	0	0	0	1	2	0	0	0	0
TGGT1_208030	microneme protein MIC4	0.65	0.5	5	0	0	11	0	0	0.61	0.48	0
TGGT1_208830	hypothetical protein	0.022	7	5	6	3	2	0	0	4.01	3.87	0.83
TGGT1_209030	actin ACT1	0.66	0.8	81	37	138	75	81	156	0.74	0.59	0
TGGT1_209755B	hypothetical protein	0.56	3	3	0	0	1	0	0	1.35	1.18	0.32
TGGT1_209910	histone H2Bv	0.8	0.7	2	0	0	3	0	0	0.87	0.8	0.08
TGGT1_210690	ribosomal protein RPS6	0.43	2.2	12	4	2	7	0	1	2.01	1.89	0.28
TGGT1_211680	protein disulfide isomerase	1	1	3	0	0	3	0	0	1	0.87	0.16
TGGT1_212290	ribosomal protein RPS19	0.67	1.6	8	2	1	6	0	1	1.41	1.31	0.2
TGGT1_213280	SAG-related sequence SRS25	0.37	0	0	0	0	6	0	0	0	0	0
TGGT1_213350	ribosomal protein RPS15	0.32	1.8	10	7	3	7	2	2	1.65	1.6	0.1
TGGT1_214080	toxofilin	0.37	INF	0	0	3	0	0	0	2.36	1.72	0.33
TGGT1_214220	hypothetical protein	0.11	0.5	7	3	4	12	9	6	0.52	0.52	0
TGGT1_214575	hypothetical protein	0.37	0	0	0	0	4	0	0	0	0	0
TGGT1_214770	putative small GTP binding protein rab1a	0.12	0	0	0	0	2	0	2	0	0	0
TGGT1_214880	hypothetical protein	0.19	0.1	2	0	0	13	7	0	0.21	0.19	0
TGGT1_214940	MIC2-associated protein M2AP	0.37	INF	3	0	0	0	0	0	1.63	1.43	0.33
TGGT1_215160	hypothetical protein	0.16	INF	1	2	0	0	0	0	1.98	1.76	0.32
TGGT1_215220	hypothetical protein	0.37	INF	2	0	0	0	0	0	1.42	1.31	0.32
TGGT1_215460	ribosomal protein RPS24	0.17	3.2	5	6	2	4	0	0	2.84	2.68	0.51
TGGT1_215470	ribosomal protein RPL10A	0.72	1.5	10	1	1	6	0	2	1.21	1.03	0.24
TGGT1_215775	rhoptry protein ROP8	0.37	0	0	0	0	0	0	1	0	0	0
TGGT1_215980	hypothetical protein	0.62	0.7	7	2	2	10	1	5	0.69	0.68	0
TGGT1_216000	alveolin domain containing intermediate filament IMC3	0.5	0.7	26	11	10	37	16	14	0.67	0.67	0
TGGT1_216180	hypothetical protein	0.8	1.5	3	0	0	2	0	0	1.15	1	0.22
TGGT1_216210	hypothetical protein	0.56	0.3	0	0	1	0	3	0	0.63	0.57	0
TGGT1_216670	FUSE-binding protein 2 / KH-type splicing regulatory protein	0.65	2	4	0	0	1	1	0	1.12	0.92	0.29
TGGT1_216770	hypothetical protein	1	INF	2	2	2	0	0	0	3.1	3.03	0.96
TGGT1_217570	ribosomal protein RPS27	0.61	0.6	4	0	0	3	4	0	0.54	0.45	0.01
TGGT1_218260	histone H3.3	0.71	1.5	3	3	0	4	0	0	1.5	1.27	0.19

TGGT1_218410	ribosomal protein RPP0	0.78	1.4	11	2	0	9	0	0	1.4	1.02	0.23
TGGT1_218520	microneme protein MIC6	0.37	0	0	0	0	2	0	0	0	0	0
TGGT1_219270	multi-pass transmembrane protein	0.013	0.3	6	2	2	11	9	9	0.34	0.33	0
TGGT1_219280	actin like protein ALP1	0.73	1.7	7	0	0	4	0	0	1.34	0.95	0.27
TGGT1_219320	acid phosphatase GAP50	0.17	0.6	35	19	17	59	37	29	0.53	0.53	0
TGGT1_219820 (+1)	putative polyubiquitin UbC	0.8	0.7	0	0	4	6	0	0	1.23	0.82	0.05
TGGT1_220240	hypothetical protein	0.094	5.5	2	6	3	2	0	0	3.57	3.16	0.68
TGGT1_220270	alveolin domain containing intermediate filament IMC6	0.48	0.7	29	11	12	35	19	17	0.67	0.66	0
TGGT1_220930	hypothetical protein	0.28	0.1	1	0	0	8	2	0	0.34	0.33	0
TGGT1_220950	hypothetical protein	0.0069	9.5	5	8	6	2	0	0	5.5	5.12	0.94
TGGT1_221470	hypothetical protein	0.37	INF	3	0	0	0	0	0	1.63	1.43	0.33
TGGT1_221620	putative beta-tubulin	0.37	0.8	49	31	27	55	40	38	0.73	0.72	0
TGGT1_221675A	hypothetical protein	0.56	0.3	0	1	0	3	0	0	0.84	0.79	0
TGGT1_222160	aldehyde dehydrogenase	0.37	0	0	0	0	0	3	0	0	0	0
TGGT1_222170	dense-granule antigen DG32	0.49	0.2	2	0	0	9	0	0	0.49	0.45	0
TGGT1_222210	SPFH domain / Band 7 family protein	0.24	0.08	1	0	0	9	3	0	0.29	0.28	0
TGGT1_222220	alveolin domain containing intermediate filament IMC7	0.026	0.5	18	16	14	39	29	25	0.5	0.49	0
TGGT1_223000	dynein light chain DLC	0.37	0	0	0	0	4	0	0	0	0	0
TGGT1_223920	rhoptry neck protein RON3	0.45	6.5	13	0	0	2	0	0	2.63	1.47	0.33
TGGT1_223940	GAP45 protein (GAP45)	0.17	0.7	12	6	8	12	12	11	0.67	0.66	0
TGGT1_224130	hypothetical protein	0.52	0.4	7	0	0	12	4	0	0.47	0.33	0
TGGT1_224850	putative polyadenylate binding protein	0.37	0	0	0	0	3	0	0	0	0	0
TGGT1_225080	ribosomal protein RPS18	0.037	3.9	11	14	6	4	3	1	3.12	2.94	0.7
TGGT1_225150	hypothetical protein	0.68	2	2	0	0	1	0	0	1.17	1.08	0.24
TGGT1_225560	hypothetical protein	0.37	0	0	0	0	3	0	0	0	0	0
TGGT1_225690	hypothetical protein	0.31	0.2	8	1	0	27	8	2	0.27	0.21	0
TGGT1_226220	alveolin domain containing intermediate filament IMC9	0.1	0.1	0	0	1	4	1	2	0.44	0.41	0
TGGT1_226240	putative bud site selection protein	0.0022	INF	3	2	2	0	0	0	3.31	3.29	0.97
TGGT1_226380	hypothetical protein	0.26	INF	5	1	0	0	0	0	2.44	2.08	0.33
TGGT1_226570	hypothetical protein	0.057	0.6	10	5	6	12	13	10	0.55	0.54	0
TGGT1_226810-DECOY	histone lysine methyltransferase SET1	0.19	6	4	1	1	0	1	0	1.86	1.82	0.33
TGGT1_226970	ribosomal protein RPS11	0.43	1.5	7	5	5	8	2	1	1.64	1.62	0.01
TGGT1_227280	dense granule protein GRA3	0.26	1.8	10	15	8	12	1	5	1.94	1.81	0.02
TGGT1_227360	ribosomal protein RPL3	0.67	1.9	14	1	0	7	0	1	1.46	0.93	0.29
TGGT1_227370-DECOY	hydrolase CocE/NonD family protein	0.68	0.5	0	0	2	0	0	4	0.65	0.52	0.04
TGGT1_227800	EF hand domain-containing protein	0.25	0	0	0	0	0	9	2	0	0	0
TGGT1_227810	rhoptry kinase family protein ROP11 (incomplete catalytic triad)	0.26	27	22	4	1	1	0	0	6.3	4.81	0.67
TGGT1_227952	14-3-3 superfamily protein	0.37	0	0	0	0	2	0	0	0	0	0
TGGT1_228170	inner membrane complex protein IMC2A	0.01	54	480	307	236	16	0	3	55.71	55.6	1
TGGT1_228470	ribosomal protein RPL15	0.49	2.2	7	4	0	5	0	0	1.94	1.51	0.35
TGGT1_229010	rhoptry neck protein RON4	0.97	1	14	12	0	20	2	3	1.13	0.69	0
TGGT1_229020-DECOY	putative cell-cycle-associated protein kinase CDK	0.37	0	0	0	0	3	0	0	0	0	0
TGGT1_229250A	ribosomal protein RPL28	0.61	1.7	3	1	1	3	0	0	1.51	1.5	0.16
TGGT1_229480	putative calcium binding protein precursor	0.033	6.5	11	10	5	4	0	0	5.09	4.97	0.84
TGGT1_229670	ribosomal protein RPS23	0.19	3.3	14	6	3	4	1	2	2.33	2.25	0.44
TGGT1_230160	hypothetical protein	0.5	0.5	9	1	0	11	7	1	0.48	0.35	0
TGGT1_230210	alveolin domain containing intermediate filament IMC10	0.38	0.6	35	16	13	52	26	21	0.6	0.6	0
TGGT1_230340	hypothetical protein	0.12	0.3	6	7	4	36	19	9	0.32	0.31	0
TGGT1_230850	hypothetical protein	0.21	0.3	9	4	0	16	22	3	0.29	0.22	0
TGGT1_230980	myosin I	0.37	0	0	0	0	8	0	0	0	0	0
TGGT1_231070	protein kinase	0.51	0.2	1	0	0	4	0	0	0.65	0.64	0
TGGT1_231080	ribosomal protein RPL38	0.4	2.2	5	1	3	4	0	0	2.05	1.93	0.32
TGGT1_231140	ribosomal protein RPS25	0.013	7	6	4	4	2	0	0	3.94	3.91	0.85
TGGT1_231160	hypothetical protein	0.52	0.7	2	2	1	4	2	1	0.82	0.81	0
TGGT1_231460	hypothetical protein	0.37	0	0	0	0	4	0	0	0	0	0
TGGT1_231630	alveolin domain containing intermediate filament IMC4	0.21	0.7	29	18	14	37	27	24	0.63	0.63	0
TGGT1_231640	alveolin domain containing intermediate filament IMC1	0.31	0.7	45	36	29	66	50	32	0.71	0.7	0
TGGT1_231850	serine-threonine phosphatase 2C (PP2C)	0.68	0.5	2	0	0	4	0	0	0.77	0.71	0.04
TGGT1_232130	hypothetical protein	0.42	0.4	8	0	0	15	2	4	0.38	0.26	0
TGGT1_232230	ribosomal protein RPL30	0.44	1.8	5	2	2	4	0	1	1.59	1.59	0.17

TGGT1_232300	ribosomal protein RPS3	0.28	1.9	20	11	6	9	8	3	1.54	1.51	0.27
TGGT1_232410	PDI family protein	0.046	0.5	18	10	12	34	23	22	0.48	0.48	0
TGGT1_232710	ribosomal protein RPS3A	0.36	1.9	30	13	13	23	3	3	2.1	2.09	0.05
TGGT1_232780	hypothetical protein	0.37	0	0	0	0	6	0	0	0	0	0
TGGT1_232830	putative vacuolar proton translocating ATPase subunit A	0.24	0	0	0	0	4	0	1	0	0	0
TGGT1_233010	putative cell-cycle-assocaited protein kinase ERK7	0.23	0.2	1	0	0	3	2	0	0.48	0.47	0
TGGT1_233100	SPFH domain / Band 7 family protein	0.8	0.7	6	0	0	9	0	0	0.78	0.58	0.03
TGGT1_233460	SAG-related sequence SRS29B (SAG1)	0.37	0.4	17	1	0	35	12	4	0.32	0.19	0
TGGT1_233480	SAG-related sequence SRS29C (SRS2)	0.53	0.3	2	0	0	7	0	0	0.57	0.53	0
TGGT1_234450	ribosomal protein RPS15A	0.64	1.3	9	4	5	9	3	2	1.29	1.27	0
TGGT1_235340	hypothetical protein	0.081	0	0	0	0	10	8	1	0	0	0
TGGT1_235380	hypothetical protein	0.17	0.2	4	0	1	18	9	2	0.23	0.21	0
TGGT1_235470	myosin A	0.34	0.8	82	53	51	113	77	58	0.71	0.7	0
TGGT1_236560	hypothetical protein	0.56	0.3	1	0	0	3	0	0	0.74	0.72	0
TGGT1_236580	Prp31-15.5k-U4 Snrna Complex family protein	0.56	0.3	2	0	0	6	0	0	0.62	0.58	0
TGGT1_236650	DEAD (Asp-Glu-Ala-Asp) box polypeptide 17	0.37	0	0	0	0	5	0	0	0	0	0
TGGT1_236950	hypothetical protein	0.18	0.3	12	4	0	25	15	7	0.31	0.22	0
TGGT1_237180	hypothetical protein	0.89	1.2	5	1	0	5	0	0	1.18	1.01	0.17
TGGT1_237820	IMC sub-compartment protein ISP2	0.37	0	0	0	0	2	0	0	0	0	0
TGGT1_238010	ribosomal protein RPL23A	0.23	2.6	6	4	3	5	0	0	2.5	2.49	0.37
TGGT1_238250	ribosomal protein RPL36	0.68	2	4	0	0	2	0	0	1.29	1.07	0.27
TGGT1_239100	ribosomal protein RPS7	0.15	4.4	23	12	5	8	0	1	3.99	3.81	0.59
TGGT1_239260	histone H4	0.92	1.1	11	3	2	7	4	4	0.87	0.82	0.01
TGGT1_239400	hypothetical protein	0.21	0.2	10	0	0	27	14	3	0.22	0.14	0
TGGT1_239740	dense granule protein GRA14	0.057	4.8	9	5	5	4	0	0	3.83	3.82	0.74
TGGT1_239760	ribosomal protein RPL22	0.44	1.9	4	4	5	7	0	0	2.27	2.15	0.1
TGGT1_240060	hypothetical protein	0.062	0.3	8	9	3	33	24	12	0.31	0.29	0
TGGT1_242330	ribosomal protein RPS5	0.0076	5	18	25	17	8	2	2	4.85	4.55	0.95
TGGT1_242790B	putative trichohyalin	0.16	0	0	0	0	2	1	0	0	0	0
TGGT1_243200	hypothetical protein	0.46	0.3	6	1	0	19	3	0	0.42	0.34	0
TGGT1_243570	ribosomal protein RPS26	0.12	3	3	3	3	3	0	0	2.53	2.46	0.48
TGGT1_243950	putative prohibitin	0.85	0.8	6	0	0	8	0	0	0.84	0.62	0.06
TGGT1_243960	nuclear transport factor 2 (ntf2) domain-containing protein	0.37	0	0	0	0	7	0	0	0	0	0
TGGT1_244110	nucleosome assembly protein (nap) protein	0.51	0.2	2	0	0	8	0	0	0.53	0.49	0
TGGT1_244250	hypothetical protein	0.067	0	0	0	0	1	1	3	0	0	0
TGGT1_244560	putative heat shock protein 90	0.21	INF	0	2	6	0	0	0	4.49	3.12	0.65
TGGT1_244880-DECOY	DNA-directed RNA polymerase I RPA1	0.19	0.2	0	1	0	4	1	1	0.5	0.46	0
TGGT1_245460	ribosomal protein RPS8	0.12	4.5	14	9	4	6	0	0	4.05	3.94	0.63
TGGT1_245490	microneme protein MIC8	0.46	0.2	1	0	0	6	0	0	0.53	0.52	0
TGGT1_245620	ribosomal-ubiquitin protein RPS27A	0.12	INF	4	1	1	0	0	0	2.68	2.62	0.33
TGGT1_245680	ribosomal protein RPL21	0.94	0.9	7	4	0	12	0	0	1.13	0.88	0
TGGT1_246940	hypothetical protein	0.37	0	0	0	0	3	0	0	0	0	0
TGGT1_246950	hypothetical protein	0.37	0	0	0	0	6	0	0	0	0	0
TGGT1_247440	hypothetical protein	0.031	7.5	5	7	3	2	0	0	4.28	4.05	0.84
TGGT1_248360	hypothetical protein	0.27	0	0	0	0	12	1	1	0	0	0
TGGT1_248390	ribosomal protein RPL26	0.75	1.5	7	2	0	6	0	0	1.42	1.15	0.22
TGGT1_248440-DECOY	hypothetical protein	0.37	0	0	0	0	0	2	0	0	0	0
TGGT1_248480	ribosomal protein RPS9	0.035	6.4	15	10	7	5	0	0	5.41	5.39	0.86
TGGT1_248630	actin-related protein ARP1	0.8	0.7	2	0	0	3	0	0	0.87	0.8	0.08
TGGT1_248700	alveolin domain containing intermediate filament IMC12	0.14	0.4	10	3	5	22	16	7	0.4	0.39	0
TGGT1_248740	hypothetical protein	0.15	0.06	1	0	0	12	5	1	0.19	0.19	0
TGGT1_249240	putative calmodulin	0.072	0.6	8	5	9	17	11	11	0.59	0.55	0
TGGT1_249240 [4]	Cluster of putative calmodulin	0.085	0.7	33	27	41	48	40	58	0.65	0.6	0
TGGT1_249570	hypothetical protein	0.21	INF	0	3	1	0	0	0	2.61	2.19	0.33
TGGT1_249850	GAP40 protein	0.33	0.6	4	3	2	9	5	2	0.64	0.64	0
TGGT1_249900	putative adenine nucleotide translocator	0.89	0.8	4	1	0	6	0	0	0.98	0.86	0.05
TGGT1_249990	hypothetical protein	0.25	4	2	0	2	1	0	0	1.92	1.68	0.48
TGGT1_250340	centrin 2	0.049	0.2	2	1	1	11	5	5	0.28	0.28	0
TGGT1_250710	microneme protein MIC10	0.063	0.4	7	6	13	27	15	17	0.49	0.43	0
TGGT1_250770	putative eukaryotic initiation factor-4A	0.71	0.7	4	1	0	4	3	0	0.7	0.62	0.01

TGGT1_250820	hypothetical protein	0.27	0.2	6	0	0	26	10	0	0.21	0.15	0
TGGT1_251540	dense granule protein GRA9	0.0037	49	21	16	12	1	0	0	14.04	13.93	1
TGGT1_251780	heat shock protein	0.37	0	0	0	0	2	0	0	0	0	0
TGGT1_251810	putative translation initiation factor eIF-5A	0.37	INF	2	0	0	0	0	0	1.42	1.31	0.32
TGGT1_252360	rhoptry kinase family protein ROP24 (incomplete catalytic triad)	0.19	8	5	3	0	1	0	0	2.65	2.18	0.65
TGGT1_253370	hypothetical protein	0.37	0	0	0	0	4	0	0	0	0	0
TGGT1_253470	alveolin domain containing intermediate filament IMC13	0.13	0	0	0	0	3	2	0	0	0	0
TGGT1_254370	guanylyl cyclase	0.56	0.3	0	0	1	3	0	0	0.89	0.81	0
TGGT1_254440	ribosomal protein RPL12	0.81	1.3	3	1	0	3	0	0	1.23	1.12	0.16
TGGT1_254470	hypothetical protein	0.012	INF	10	14	6	0	0	0	11.22	10.49	1
TGGT1_254720	dense granule protein GRA8	0.46	1.6	4	8	2	5	2	2	1.49	1.31	0.13
TGGT1_255190	myosin C	0.24	0	0	0	0	12	3	0	0	0	0
TGGT1_255260	apical membrane antigen AMA1	1	1	2	0	0	2	0	0	1	0.92	0.14
TGGT1_257340	Ras family protein	1	INF	0	0	0	0	0	0	0.39	0.38	0
TGGT1_257530	transporter, major facilitator family protein	0.54	0.3	3	0	0	10	0	0	0.52	0.45	0
TGGT1_257680	myosin light chain MLC1	0.45	0.7	23	12	17	36	19	15	0.75	0.73	0
TGGT1_258390	putative DnaJ protein	0.56	3	3	0	0	1	0	0	1.35	1.18	0.32
TGGT1_258410	photosensitized INA-labeled protein PHIL1	0.68	0.9	20	11	9	20	16	10	0.79	0.79	0
TGGT1_258458	hypothetical protein	0.033	10	3	5	2	0	0	1	3	2.81	0.89
TGGT1_258462	hypothetical protein	0.13	INF	2	3	0	0	0	0	2.58	2.16	0.65
TGGT1_258470	hypothetical protein	0.22	0.4	7	3	3	19	6	7	0.45	0.45	0
TGGT1_258580	rhoptry protein ROP17	0.72	1.3	23	8	6	18	8	3	1.19	1.15	0
TGGT1_258870A	hypothetical protein	0.0034	17	13	12	8	2	0	0	8.4	8.25	0.99
TGGT1_258870B	hypothetical protein	0.14	3.6	12	9	4	7	0	0	3.53	3.44	0.55
TGGT1_259010	putative vacuolar ATP synthase subunit d	0.053	0	0	0	0	10	2	7	0	0	0
TGGT1_259630	hypothetical protein	0.14	0.5	3	1	1	5	4	2	0.51	0.51	0
TGGT1_260190	microneme protein MIC13	0.51	0.2	0	0	1	4	0	0	0.78	0.72	0
TGGT1_260260	ribosomal protein RPP1	0.51	2.5	4	0	1	2	0	0	1.61	1.42	0.27
TGGT1_260540	alveolin domain containing intermediate filament IMC14	0.18	0.3	13	3	0	24	14	8	0.3	0.21	0
TGGT1_260820	IMC sub-compartment protein ISP1	0.68	0.5	2	0	0	4	0	0	0.77	0.71	0.04
TGGT1_261240	histone H3	0.48	1.4	7	8	5	8	0	6	1.39	1.34	0
TGGT1_261240 [7]	Cluster of histone H3	0.88	1.1	19	13	12	20	2	19	1	1	0
TGGT1_261250	histone H2A1	0.71	1.3	5	3	6	8	0	3	1.42	1.33	0
TGGT1_261250 [2]	Cluster of histone H2A1	0.74	1.2	6	3	6	9	0	3	1.4	1.33	0
TGGT1_261400	hypothetical protein	0.37	0	0	0	0	2	0	0	0	0	0
TGGT1_261570	ribosomal protein RPL7A	0.7	0.6	4	1	0	9	0	0	0.76	0.67	0
TGGT1_261580	histone H2AX	0.56	0.3	1	0	0	3	0	0	0.74	0.72	0
TGGT1_262050	rhoptry kinase family protein ROP39	0.1	20	23	13	3	2	0	0	8.57	7.58	0.88
TGGT1_262150	kelch repeat and K+ channel tetramerisation domain containing protein	0.16	0	0	0	0	2	1	0	0	0	0
TGGT1_262620	RNA recognition motif-containing protein	0.68	2	6	0	0	3	0	0	1.38	1.03	0.28
TGGT1_262670	ribosomal protein RPL18A	0.78	1.4	8	2	0	7	0	0	1.39	1.09	0.21
TGGT1_262690	ribosomal protein RPL27	0.72	1.5	4	2	0	4	0	0	1.41	1.22	0.2
TGGT1_262730	rhoptry protein ROP16	0.37	INF	3	0	0	0	0	0	1.63	1.43	0.33
TGGT1_262960	putative U1 snRNP-associated protein Usp106	0.00026	INF	13	17	14	0	0	0	16.64	15.64	1
TGGT1_263040	ribosomal protein RPS16	0.041	1.9	9	9	9	7	5	2	1.84	1.77	0.01
TGGT1_263050	ribosomal protein RPL13	0.86	1.3	8	1	0	7	0	0	1.23	0.94	0.21
TGGT1_263090	14-3-3 protein	0.62	0.6	15	0	2	17	5	5	0.55	0.37	0
TGGT1_263090 [8]	Cluster of 14-3-3 protein	0.12	0.4	20	1	4	24	18	19	0.32	0.25	0
TGGT1_263180	myosin D	0.37	0	0	0	0	3	0	0	0	0	0
TGGT1_263300	eukaryotic porin protein	0.82	0.7	10	0	0	13	0	1	0.73	0.46	0.01
TGGT1_263520	microtubule associated protein SPM1	0.16	0.4	19	5	1	32	20	13	0.32	0.26	0
TGGT1_263700	ribosomal protein RPS14	0.38	1.9	35	10	10	15	9	5	1.58	1.53	0.32
TGGT1_264660	SAG-related sequence SRS44	0.14	2.3	55	91	55	63	9	14	2.87	2.62	0
TGGT1_266070	ribosomal protein RPL31	0.0011	13	5	4	4	1	0	0	4.46	4.39	1
TGGT1_266080	hypothetical protein	0.13	0.2	2	1	0	12	6	2	0.25	0.24	0
TGGT1_266960	beta-tubulin	0.29	0.8	46	32	33	59	44	37	0.74	0.73	0
TGGT1_266960 [12]	Cluster of beta-tubulin	0.0033	0.7	101	87	88	119	125	125	0.67	0.65	0
TGGT1_266970	hypothetical protein	0.37	INF	2	0	0	0	0	0	1.42	1.31	0.32
TGGT1_267400	ribosomal protein RPL32	0.034	5.2	9	11	6	5	0	0	4.77	4.57	0.8
TGGT1_267500	hypothetical protein	0.35	0.4	13	2	2	25	11	4	0.41	0.37	0

TGGT1_267740	hypothetical protein	0.43	3.5	0	6	1	0	1	1	1.95	1.38	0.33
TGGT1_268560-DECOY	XPG N-terminal domain-containing protein	1	1	0	1	1	0	2	0	0.98	0.91	0
TGGT1_268850	enolase 2	0.8	0.7	4	0	0	6	0	0	0.81	0.67	0.05
TGGT1_269180	MIF4G domain-containing protein	0.56	3	3	0	0	0	1	0	1.13	0.99	0.32
TGGT1_269190	glyceraldehyde-3-phosphate dehydrogenase GAPDH2	0.68	0.5	2	0	0	4	0	0	0.77	0.71	0.04
TGGT1_269442	putative calmodulin	0.099	0.6	6	3	5	9	7	6	0.64	0.62	0
TGGT1_269885A	rhoptry metalloprotease toxolysin TLN1	0.51	0.2	1	0	0	4	0	0	0.65	0.64	0
TGGT1_269885B	rhoptry metalloprotease toxolysin TLN1	0.8	0.7	2	0	0	3	0	0	0.87	0.8	0.08
TGGT1_269950	hypothetical protein	0.3	INF	8	1	0	0	0	0	3.08	2.36	0.33
TGGT1_269980	putative preprotein translocase Sec61	0.46	0.2	1	0	0	6	0	0	0.53	0.52	0
TGGT1_270240	MAG1 protein (MAG1)	0.0034	3.2	34	44	34	15	7	13	2.97	2.79	0.94
TGGT1_270250	dense granule protein GRA1	0.37	3.1	18	5	2	8	0	0	2.82	2.51	0.34
TGGT1_270320	protein phosphatase 2C domain-containing protein	0.041	INF	7	4	2	0	0	0	4.93	4.85	0.99
TGGT1_270380	ribosomal protein RPS13	0.14	3.8	8	4	3	4	0	0	3.02	3.01	0.55
TGGT1_270690	arginyl-tRNA synthetase	0.37	0	0	0	0	11	0	0	0	0	0
TGGT1_270720	hypothetical protein	0.16	0	0	0	0	1	0	2	0	0	0
TGGT1_271050	SAG-related sequence SRS34A (SAG2A)	0.57	0.5	8	0	0	12	3	1	0.5	0.34	0
TGGT1_271930	hypothetical protein	0.26	0.6	11	4	3	10	13	7	0.51	0.5	0
TGGT1_271935	hypothetical protein	0.065	0.1	1	0	0	5	2	2	0.31	0.3	0
TGGT1_271970	glideosome-associated protein with multiple-membrane spans GAPM3	0.059	0.5	3	7	2	8	8	8	0.52	0.45	0
TGGT1_271980-DECOY	SAG-related sequence SRS32	0.37	0	0	0	0	0	2	0	0	0	0
TGGT1_272030A	kelch repeat-containing protein	0.37	0	0	0	0	6	0	0	0	0	0
TGGT1_272520	hypothetical protein	0.3	0.2	3	0	0	9	4	0	0.35	0.3	0
TGGT1_272600	adaptin c-terminal domain-containing protein	0.37	0	0	0	0	3	0	0	0	0	0
TGGT1_273560	putative kinesin heavy chain	0.4	0.3	7	0	0	20	5	0	0.33	0.24	0
TGGT1_273760	heat shock protein HSP70	0.66	0.7	20	7	4	34	8	4	0.71	0.68	0
TGGT1_273760 [6]	Cluster of heat shock protein HSP70	0.3	0.5	20	7	9	37	9	22	0.51	0.5	0
TGGT1_273860	hypothetical protein	0.17	0.1	2	0	0	9	7	0	0.24	0.22	0
TGGT1_275440	dense granule protein GRA6	0.37	INF	0	2	0	0	0	0	1.77	1.49	0.32
TGGT1_275810	ribosomal protein RPS10	0.81	0.8	5	1	0	7	0	1	0.82	0.7	0.02
TGGT1_275860	hypothetical protein	0.37	INF	2	0	0	0	0	0	1.42	1.31	0.32
TGGT1_276140	ADP ribosylation factor ARF1	0.35	0.3	3	0	0	8	3	0	0.41	0.35	0
TGGT1_277270	NTPase II	0.88	1.2	5	0	0	4	0	0	1.11	0.87	0.22
TGGT1_278130	hypothetical protein	0.37	0	0	0	0	4	0	0	0	0	0
TGGT1_278205-DECOY	hypothetical protein	0.37	INF	0	2	0	0	0	0	1.77	1.49	0.32
TGGT1_278870	myosin F	0.22	0.5	94	41	23	142	86	65	0.47	0.45	0
TGGT1_278990	putative phosphate carrier	0.56	0.3	1	0	0	3	0	0	0.74	0.72	0
TGGT1_279100	hypothetical protein	0.01	INF	9	7	4	0	0	0	7.41	7.32	1
TGGT1_280560	selenide, water dikinase	0.76	0.7	4	0	0	5	0	1	0.72	0.6	0.04
TGGT1_280660	HECT-domain (ubiquitin-transferase) domain-containing protein	0.78	1.2	1	3	1	0	1	3	0.97	0.89	0.07
TGGT1_282170	hypothetical protein	0.16	INF	2	1	0	0	0	0	1.81	1.7	0.32
TGGT1_284560	ribosomal protein RPL9	0.6	1.8	7	1	1	4	0	1	1.42	1.29	0.25
TGGT1_286420A	putative elongation factor 1-alpha (EF-1-ALPHA)	0.77	0.8	24	7	3	25	12	5	0.72	0.65	0
TGGT1_286450	dense granule protein GRA5	0.33	2.5	5	4	1	4	0	0	2.18	2.05	0.39
TGGT1_286580	hypothetical protein	0.28	0.5	45	13	9	74	43	21	0.43	0.4	0
TGGT1_286590	microtubule associated protein SPM2	0.31	0.4	9	2	0	11	13	2	0.37	0.28	0
TGGT1_286600	hypothetical protein	0.24	0.3	6	0	0	13	5	3	0.3	0.23	0
TGGT1_288360	tryptophanyl-tRNA synthetase (TrpRS2)	0.58	0.5	2	0	0	3	1	0	0.68	0.63	0.02
TGGT1_288380	heat shock protein HSP90	0.78	1.2	7	3	0	1	4	3	0.82	0.65	0.12
TGGT1_288650	dense granule protein GRA12	0.95	1	29	21	14	48	14	5	1.09	1.08	0
TGGT1_288720	ribosomal protein RPL10	0.2	3.6	10	7	1	3	2	0	2.48	2.14	0.63
TGGT1_289530	ribosomal protein RPL19	0.8	1.3	5	3	0	6	0	0	1.41	1.16	0.13
TGGT1_289690	glyceraldehyde-3-phosphate dehydrogenase GAPDH1	0.72	0.6	5	0	0	9	0	0	0.7	0.55	0.01
TGGT1_289970	hypothetical protein	0.35	5	4	0	1	1	0	0	1.89	1.67	0.33
TGGT1_290670	leucyl aminopeptidase LAP	0.68	0.5	1	0	0	2	0	0	0.85	0.83	0
TGGT1_290700	hypothetical protein	0.057	15	2	8	5	1	0	0	5.58	4.66	0.91
TGGT1_290920	oxidoreductase, 2OG-Fe(II) oxygenase family protein	0.37	0	0	0	0	4	0	0	0	0	0
TGGT1_291850	ribosomal protein RPS30	0.12	INF	0	2	2	0	0	0	2.68	2.31	0.64
TGGT1_291890	microneme protein MIC1	0.58	0.5	6	0	0	11	2	0	0.54	0.4	0
TGGT1_292130	ribosomal protein RPL13A	0.71	1.6	7	0	1	4	0	1	1.25	1	0.25

TGGT1_292950	hypothetical protein	0.12	0	0	0	0	2	2	0	0	0	0
TGGT1_293190	endonuclease/exonuclease/phosphatase family protein	0.23	0.2	1	0	0	2	3	0	0.44	0.43	0
TGGT1_293360	hypothetical protein	0.12	0.4	10	4	3	19	10	10	0.42	0.41	0
TGGT1_294200	glucose-6-phosphate 1-dehydrogenase	0.16	INF	0	1	2	0	0	0	2.29	2	0.32
TGGT1_294790	hypothetical protein	0.37	0	0	0	0	2	0	0	0	0	0
TGGT1_295110	rhoptry protein ROP7	0.52	3.2	18	1	0	6	0	0	2.28	1.31	0.32
TGGT1_295110 [2]	Cluster of rhoptry protein ROP7	0.59	2.4	21	1	0	9	0	0	1.99	1.07	0.31
TGGT1_295125	rhoptry protein ROP4	1	1	6	0	0	6	0	0	0.99	0.74	0.17
TGGT1_295360	hypothetical protein	0.35	0.6	14	3	3	17	11	7	0.5	0.47	0
TGGT1_297520	proteophosphoglycan PPG1	0.35	0.2	1	0	0	3	0	1	0.57	0.55	0
TGGT1_297880	dense granule protein DG32	0.18	3.1	10	11	4	8	0	0	3.38	3.19	0.51
TGGT1_299050	ribosomal protein RPL17	0.1	6.3	7	10	2	3	0	0	4.41	3.86	0.7
TGGT1_299780	hypothetical protein	0.21	14	10	4	0	1	0	0	3.84	2.85	0.67
TGGT1_300000	ribosomal protein RPL18	0.57	2	5	1	0	2	1	0	1.31	1.11	0.29
TGGT1_300100	rhoptry neck protein RON2	0.41	0.4	14	0	0	25	4	7	0.34	0.19	0
TGGT1_300190	ribosomal protein RPL37A	0.091	INF	2	5	1	0	0	0	3.8	3.31	0.65
TGGT1_300200	histone H2AZ	0.25	2	5	3	2	3	0	2	1.57	1.57	0.2
TGGT1_304670	leucine rich repeat-containing protein	0.37	0	0	0	0	4	0	0	0	0	0
TGGT1_304955	serine/threonine specific protein phosphatase	0.057	31	17	10	4	1	0	0	8.45	8.11	1
TGGT1_305050	putative calmodulin	0.57	0.7	14	2	4	16	6	7	0.62	0.57	0
TGGT1_305160	histone H2Ba	0.39	3	7	2	0	1	1	1	1.52	1.22	0.38
TGGT1_305520	ribosomal protein RPS2	0.88	1.2	18	2	2	12	4	3	0.96	0.79	0.06
TGGT1_306060	rhoptry neck protein RON8	0.51	0.3	9	0	0	31	1	0	0.36	0.23	0
TGGT1_308020	SAG-related sequence SRS57 (SAG3)	0.37	0	0	0	0	6	0	0	0	0	0
TGGT1_308090	rhoptry protein ROP5	0.29	1.8	53	33	16	32	15	9	1.71	1.66	0
TGGT1_308090 [2]	Cluster of rhoptry protein ROP5	0.31	1.9	57	33	16	33	15	9	1.73	1.69	0
TGGT1_308840	SAG-related sequence SRS51 (SRS3)	0.51	0.2	1	0	0	4	0	0	0.65	0.64	0
TGGT1_308860	hypothetical protein	0.1	0.4	14	9	4	29	18	14	0.42	0.41	0
TGGT1_309120	ribosomal protein RPL4	0.5	2	19	7	2	12	1	1	1.92	1.69	0.27
TGGT1_309590	rhoptry protein ROP1	0.72	1.1	4	6	6	6	4	5	1.06	0.98	0
TGGT1_309760	hypothetical protein	0.18	INF	2	5	0	0	0	0	3.35	2.49	0.65
TGGT1_309810	ribosomal protein RPP2	0.76	1.4	8	0	2	6	0	1	1.3	1.02	0.2
TGGT1_309820	ribosomal protein RPL11	0.067	INF	9	3	3	0	0	0	5.42	5.34	1
TGGT1_310010	rhoptry neck protein RON1	0.59	0.5	4	1	0	10	0	1	0.62	0.54	0
TGGT1_310070	putative methyltransferase	0.48	0.2	1	0	0	5	0	0	0.59	0.57	0
TGGT1_310320	calreticulin family protein	0.37	INF	4	0	0	0	0	0	1.85	1.52	0.33
TGGT1_310490	ribosomal protein RPL27A	0.15	3.5	3	1	3	2	0	0	2.37	2.22	0.43
TGGT1_310780	dense granule protein GRA4	0.72	1.2	8	9	4	12	3	2	1.36	1.3	0
TGGT1_311230	hypothetical protein	0.0096	0.05	3	0	0	27	22	13	0.07	0.06	0
TGGT1_311240	putative DnaJ family chaperone	0.84	1.3	12	0	0	8	0	1	1.11	0.64	0.26
TGGT1_311290	protein tyrosine phosphatase family protein, ptpla protein	0.68	2	2	0	0	1	0	0	1.17	1.08	0.24
TGGT1_311470	rhoptry neck protein RON5	0.36	0.4	17	6	0	32	15	5	0.41	0.27	0
TGGT1_311720	chaperonin protein BiP	0.018	5.4	65	34	41	13	10	3	4.81	4.76	0.99
TGGT1_312090	ribosomal protein RPL23	0.53	2.2	19	5	0	9	2	0	1.83	1.17	0.3
TGGT1_312270	rhoptry protein ROP13	0.8	1.4	9	1	0	7	0	0	1.32	0.98	0.24
TGGT1_313380	hypothetical protein	0.18	0.6	21	15	10	37	26	16	0.56	0.56	0
TGGT1_313390	ribosomal protein RPL6	0.6	2.6	13	0	0	5	0	0	1.81	1.02	0.31
TGGT1_313440	hypothetical protein	0.16	INF	2	1	0	0	0	0	1.81	1.7	0.32
TGGT1_313480	hypothetical protein	0.3	0.4	4	0	0	6	4	1	0.41	0.34	0
TGGT1_313560	putative 60S ribosomal protein L7a	0.56	3	6	0	0	2	0	0	1.59	1.18	0.32
TGGT1_314080	hypothetical protein	0.37	0.4	3	0	0	4	4	0	0.45	0.39	0
TGGT1_314810	ribosomal protein RPL7	0.33	3.2	13	4	2	6	0	0	2.72	2.53	0.36
TGGT1_315320	SAG-related sequence SRS52A	0.43	0.1	1	0	0	9	0	0	0.41	0.4	0
TGGT1_315610	hypothetical protein	0.0052	21	5	9	7	1	0	0	7.16	6.54	1
TGGT1_315750	hypothetical protein	1	1	2	1	0	2	1	0	0.97	0.91	0.05
TGGT1_316250	hypothetical protein	0.0056	INF	50	34	27	0	0	0	36.9	36.77	1
TGGT1_316340	hypothetical protein	0.41	0.5	6	2	0	9	6	1	0.5	0.42	0
TGGT1_316400B	alpha tubulin TUBA1	0.26	0.7	44	28	22	60	38	36	0.65	0.64	0
TGGT1_316400B [6]	Cluster of alpha tubulin TUBA1	0.066	0.7	73	54	50	94	74	79	0.65	0.64	0
TGGT1_318160	MSP (Major sperm protein) domain-containing protein	0.21	0	0	0	0	6	0	2	0	0	0

TGGT1_318525	hypothetical protein	0.61	0.4	2	0	0	5	0	0	0.69	0.64	0.01
TGGT1_319340	hypothetical protein	0.0041	INF	8	12	7	0	0	0	10.48	9.83	1
TGGT1_319560	microneme protein MIC3	0.093	0.5	22	9	6	36	23	22	0.4	0.39	0
TGGT1_320050	ribosomal protein RPL5	0.37	INF	5	0	0	0	0	0	2.06	1.61	0.33
TGGT1_321500	RNA recognition motif-containing protein	0.21	0	0	0	0	3	0	1	0	0	0
TGGT1_324600	heat shock protein	0.18	0.6	29	14	9	37	31	21	0.51	0.5	0
TGGT1_360830	nucleoside-triphosphatase	0.8	1.5	6	0	0	4	0	0	1.22	0.91	0.25
TGGT1_360830 [2]	Cluster of nucleoside-triphosphatase	0.9	1.2	6	0	0	5	0	0	1.1	0.82	0.22
TGGT1_363030	rhoptry protein ROP8	0.19	3.9	31	15	5	9	0	4	3.2	2.94	0.54
TGGT1_363030 [2]	Cluster of rhoptry protein ROP8	0.2	3.6	31	15	5	9	0	5	2.91	2.68	0.5
TGGT1_410360	putative transmembrane protein	0.04	9.5	7	9	3	2	0	0	5.12	4.77	0.87
TGGT1_410370	putative transmembrane protein	0.035	5	9	7	4	3	1	0	3.56	3.52	0.77
TGGT1_410370 [3]	Cluster of putative transmembrane protein	0.037	5.7	10	9	4	3	1	0	4.03	3.91	0.8
TGGT1_411430	rhoptry protein ROP5	0.44	1.6	31	17	7	18	10	7	1.36	1.3	0.01
TGGT1_411470	putative transmembrane protein	0.37	INF	6	0	0	0	0	0	2.27	1.69	0.33
TGGT1_411760	actin	0.54	0.8	141	83	219	145	146	264	0.72	0.61	0
