# Supplementary material for: The Secreted Acid Phosphatase Domain-Containing GRA44 from Toxoplasma gondii Is Required for c-Myc Induction in Infected Cells
Source: mSphere. 2020 Feb 19;5(1):e00877-19. doi: 10.1128/mSphere.00877-19 (PMC7031617; doi:10.1128/mSphere.00877-19)
Supplement: FIG S3 [file mSphere.00877-19-sf003.pdf]

```
                                                                     *
GRA44 PD          YPSMKF-PRCTMLKCVMQD----TWSKNAVETITVEKKDTNSLKLLLIGNTGIGEYKSRA
Soybean_PAP       MGTQRSKPSCTIVAIFLAFCCFVSSSKAKLESLQHAPKADGSLSFLVVGDWG--------
Human_ACP5        MDM-------WTALLILQA-LLLPSLAD-------GATP--ALRFVAVGDWG-------G
Arabidopsis_PAP8  MGK---------------------------------------------------------
PIG_ACP5          MDT-------WTVLLILQASLVLPGAVGTRTNTRTAPTP--ILRFVAVGDWG-------G
                                                           =  *
GRA44 PD          ERKGLWYKLKRFLWTNEFDQTVSALAKWHAEEKADAVLGLGDFLGIPGPLSARDERFTKR
Soybean_PAP       -RKGAYNQS---LVAFQMGVIGEKLD-------VDFVISTGDNFYDNGLTGVFDPSFEES
Human_ACP5        VPNAPFHTAREMANAKEIARTVQILG-------ADFILSLGDNFYFTGVQDINDKRFQET
Arabidopsis_PAP8  --------------------IGKDLN-------IDFLISTGDNFYDDGIISPYDSQFQDS
PIG_ACP5          VPNAPFHTAREMANAKAIATTVKTLG-------ADFILSLGDNFYFTGVHDAKDKRFQET
                                      #
GRA44 PD          WYDIFVKDAKLDIPWLMTLGEEEALVNPSASVRHHYTGEHPNWYMPNDAYTATFSFSTSM
Soybean_PAP       FTKIYTAPSLQK-KWYNVLGNHDYRGNAKAQISHVLRYRDNRWVCFRSY-----------
Human_ACP5        FEDVFSDRSLRKVPWYVLAGNHDHLGNVSAQIA--YSKISKRWNFPSPFYRLHFK----I
Arabidopsis_PAP8  FTNIYTATSLQK-PWYNVLGNHDYRGNVYAQLSPILRDLDCRWICLRSY-----------
PIG_ACP5          FEDVFSDPSLRNVPWHVLAGNHDHLGNVSAQIA--YSKISKRWNFPSPYYRLRFK----I

GRA44 PD          TMANGTIQHEAFNATVINVNTWNLFVGNPIAN-NMQSMMDRLMWLSDQL----YTAVNQT
Soybean_PAP       TLNSENVDFFFVDTTPY---VDKYFIEDKGHNYDWRGILPRKRYTSNLLKDVDLALRQST
Human_ACP5        PQTNVSVAIFMLDTVTLCGNSDDFLSQQPERP--RDVKLARTQ-----LSWLKKQLAAAR
Arabidopsis_PAP8  VVNAEIVDIFFVDTTPF---VDRYFDEPKDHVYDWRGVLPRNKYLNSLLTDVDVALQESM
PIG_ACP5          PRSNVSVAIFMLDTVTLCGNSDDFVSQQPERP--RNLALARTQ-----LAWIKKQLAAAK
                          #                                                   #
GRA44 PD          TNWLIIMGHLPLVSTGPQGEQGRLQYVDDLYKNGQPRGPEAVLIQMLLSHYQVDLYVSAH
Soybean_PAP       ATWKVVIGHHTIKNIGHHGD------TQELLIHFLP----------LLKANNVDLYMNGH
Human_ACP5        EDYVLVAGHYPVWSIAEHGP------THCLVKQLRP----------LLATYGVTAYLCGH
Arabidopsis_PAP8  AKWKIVVGHHTIKSAGHHGN------TIELEKQLLP----------ILEANEVDLYINGH
PIG_ACP5          EDYVLVAGHYPVWSIAEHGP------THCLVKQLLP----------LLTTHKVTAYLCGH
                   *
GRA44 PD          DHFMEYVALEDLSKNTTTAFITSGAA----------------------------------V
Soybean_PAP       DHCLEHIS----SLDSSVQFLTSGGGS--KAWRGDTKQSEGDEMKFYYDGQ----GFMSV
Human_ACP5        DHNLQYLQ----D-ENGVGYVLSGAGNFMDPSKRHQRKVPNGYLRFHYGTEDSLGGFAYV
Arabidopsis_PAP8  DHCLEHIS----SINSGIQFMTSGGGS--KAWKGDVNDWNPQEMRFYYDGQ----GFMSV
PIG_ACP5          DHNLQYLQ----D-ENGLGFVLSGAGNFMDPSKKHLRKVPNGYLRFHFGAENSLGGFAYV

GRA44 PD          RLLDKDVGRGWIGRLRGALYPI-LCWSGRR--ILYAF
Soybean_PAP       HISQTQLRISFFDVFGNAIHK----WNTCKFDS-SDM
Human_ACP5        EISSKEMTVTYIEASGKSLFKTRLPRRARP
Arabidopsis_PAP8  YTSEAELRVVFYDGLGHVLHR----WSTLKNGVYSDI
PIG_ACP5          EITPKEMSVTYIEASGKSLFKTKLPRRARSEHQHRRA
```
