# Supplementary material for: The Secreted Acid Phosphatase Domain-Containing GRA44 from Toxoplasma gondii Is Required for c-Myc Induction in Infected Cells
Source: mSphere. 2020 Feb 19;5(1):e00877-19. doi: 10.1128/mSphere.00877-19 (PMC7031617; doi:10.1128/mSphere.00877-19)
Supplement: TABLE S3 [file mSphere.00877-19-st003.pdf]

Primer Use	Primer #	Primer Name	Sequence
Cloning Gra44 C-terminal region into endogenous tagging vector	1	IMC2A-HA-LIC.For	ttccaatccaatttaattaaagacagcacgggaacttgc
	2	IMC2A-HA-LIC.Rev	ccacttccaattttaattaattccgttgcgtcagtcg
Inserting Gra44 gDNA to pTNRLUC-HX	3	IMC2A-TNRLUC.FOR	TTTCGACAAAccATGGAAAGACGTACACGTCCG
	4	IMC2A-TNRLUC.REV	CGGGCTTGCGGTttaattaaTTAGGCATAATCTGGAACATCGTAAGGATAttc cgttgcgtcagtcgagtc
Amplification of TATi cassette for insertion at Gra44 promoter	5	TATi-IMC2A.F	GCTCGGTATTCATACCTAGAGATTCCTCTGTTTTCGAACAAttctcatgtttgcg gatccg
	6	TATi-IMC2A.R	CCGGACGTGTACGTCTTTCCATCTTTGTAGAAACGTGGGCcaggtcctcctcg gagatga
Q5 insertion of Gra44 promoter sgRNA to Cas9 plasmid	7	TATi-IMC2A sgRNA.F	tcattgtcccGTTTTAGAGCTAGAAATAGC
	8	TATi-IMC2A sgRNA.R	tcgtccccgcAACTTGACATCCCCATTTAC
Insertion and tagging of Gra44 gene to complement vector	9	IMC2A-myc CmR.F	ACGGGAATTCCCTAGATTGGGTACCGGGCCC
	10	IMC2A-myc CmR.R	CAACTTTTCTACATATTACAGGTCCTCCTCGGAGATCAGCTTCTGCTCttc cgttgcgtcagtcgag
Amplification of Gra44 complement cassette for Ku80 site insertion	11	IMC2A-CmR [Ku80].F	GTCCCCGGTTCGCCTCAGCACACACACACATGACGTACATCGAAGCTG GGTACCCTGTACTTCC
	12	IMC2A-CmR [Ku80].R	GTAATGTCGGAATAGTTCCCATCAGAAACAATGGAGCTATCCGCGTCCC ATTCGCCATTCAG
Insertion of Ku80 sgRNA to Cas9 plasmid	13	sgKU80.F1	ctcatattccGTTTTAGAGCTAGAAATAGC
	14	sgKU80.R1	aaaggtgtacAACTTGACATCCCCATTTAC
Myc epitope insertion upstream of TEXEL1	15	myc-PEXEL1.F	tccgaggaggacctgCGGAGAGAGCTAGAGGAAC
	16	myc-PEXEL1.R	gatcagcttctgctcCAGTCCGCCAATCGATCGCTT
TEXEL1 deletion	17	PEXEL1_delete.F	CTCACAGAGAAAGTAAAAGTAGAGTTGCGTGA
	18	PEXEL1_delete.R	CCGCAGTCCGCCAATCGATC
Gra44 R1205A mutant generation (TEXEL1)	19	Quikchange R1205A.F	gattggcggactgcgggcagagctagaggaactc
	20	Quikchange R1205A.R	gagttcctctagctctgcccgcagtccgccaatc
Gra44 L1207 mutant generation (TEXEL1)	21	Quikchange L1207A.F	cggactgcggagagaggcagaggaactcacagag
	22	Quikchange L1207A.R	ctctgtgagttcctctgcctctctccgcagtccg
Gra44 E1209 mutant generation (TEXEL1)	23	Quikchange E1209A.F	gcggagagagctagaggcactcacagagaaagtaa
	24	Quikchange E1209A.R	ttactttctctgtgagtgcctctagctctctccgc
TEXEL2 deletion	25	PEXEL2_delete.F	TTGTTTGAACCGAAGAAAGAACCAAC
	26	PEXEL2_delete.R	CGAACCACTTCCTGCACG
Gra44 R1348A mutant generation (TEXEL2)	27	IMC2A-R1348A.F	AAGTGGTTCGgcCCGGCTCTTG
	28	IMC2A-R1348X.R	CCTGCACGTTTCTCCTCAGG
Gra44 L1350A mutant generation (TEXEL2)	29	IMC2A-L1350A.F	TTCGCGCCGGgcCTTGGAATT
	30	IMC2A-L1350X.R	CCACTTCCTGCACGTTTCTCCTC
Gra44 L1352A mutant generation (TEXEL2)	31	IMC2A E1352A.F	ccggctcttggcattgtttg
	32	IMC2A E1352X.R	cgcgaaccacttcctgc
Cloning TGGT1_316250 C-terminal region into endogenous tagging vector	33	316250-myc.FOR	ttccaatccaatttaactgtgctaagccgttctagcgt
	34	316250-myc.REV	ccacttccaattttatactgttccttagccatcatgtcgag

Table S3
