# Supplementary material for: The Secreted Acid Phosphatase Domain-Containing GRA44 from Toxoplasma gondii Is Required for c-Myc Induction in Infected Cells
Source: mSphere. 2020 Feb 19;5(1):e00877-19. doi: 10.1128/mSphere.00877-19 (PMC7031617; doi:10.1128/mSphere.00877-19)
Supplement: DATA SET S2 [file mSphere.00877-19-sd002.pdf]

For figure 1B

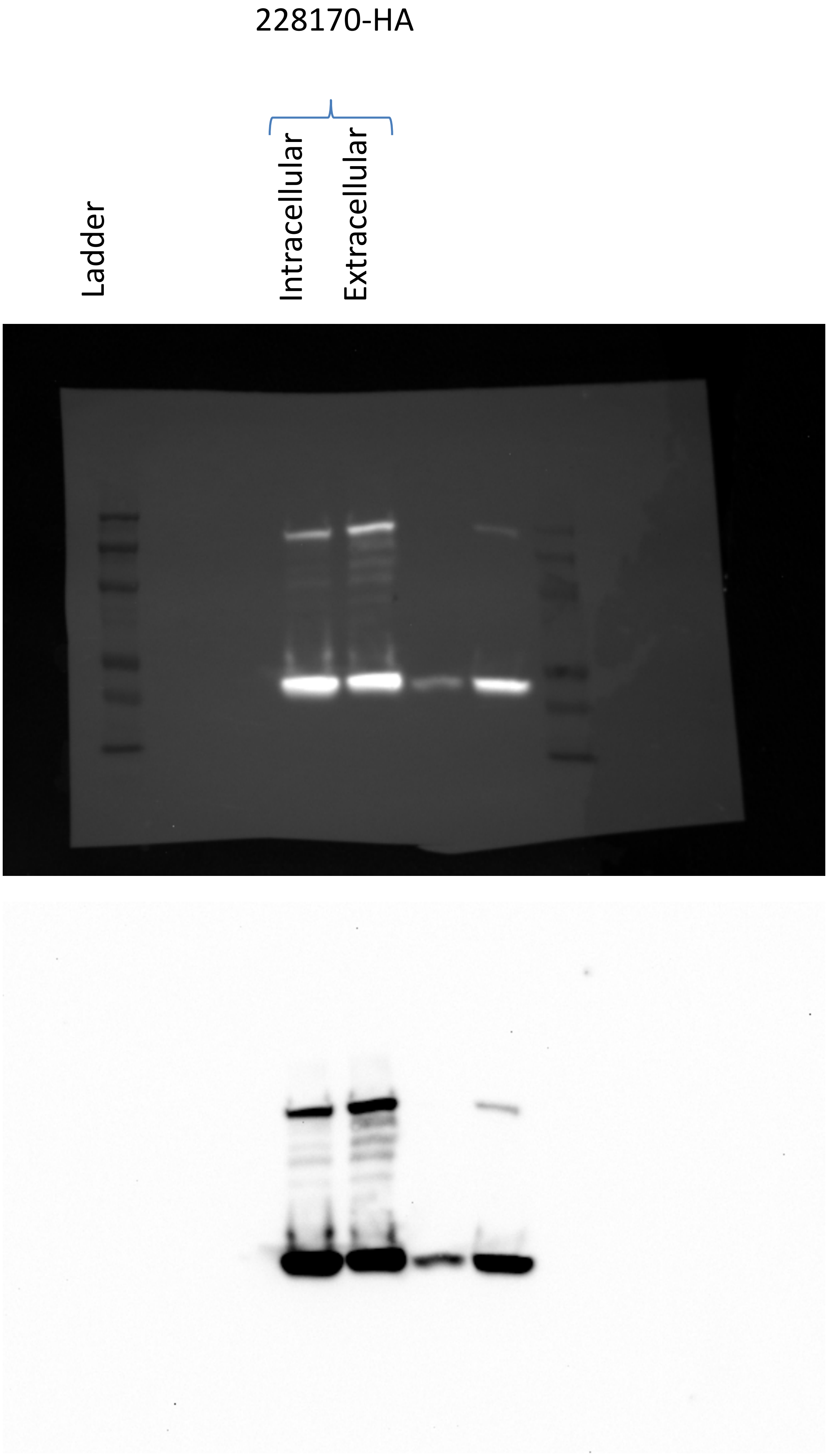

For figure 2B

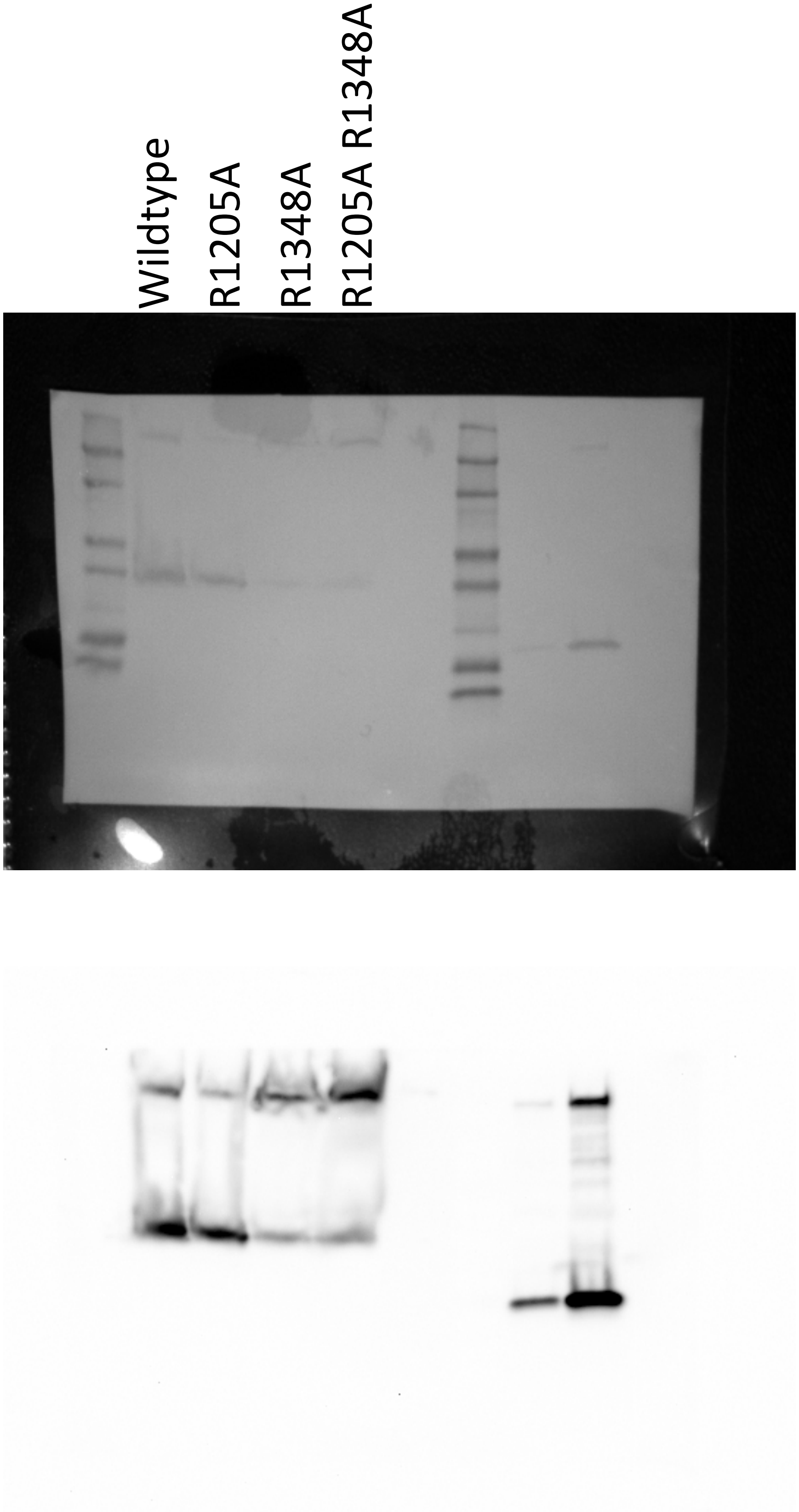

For figure 2C

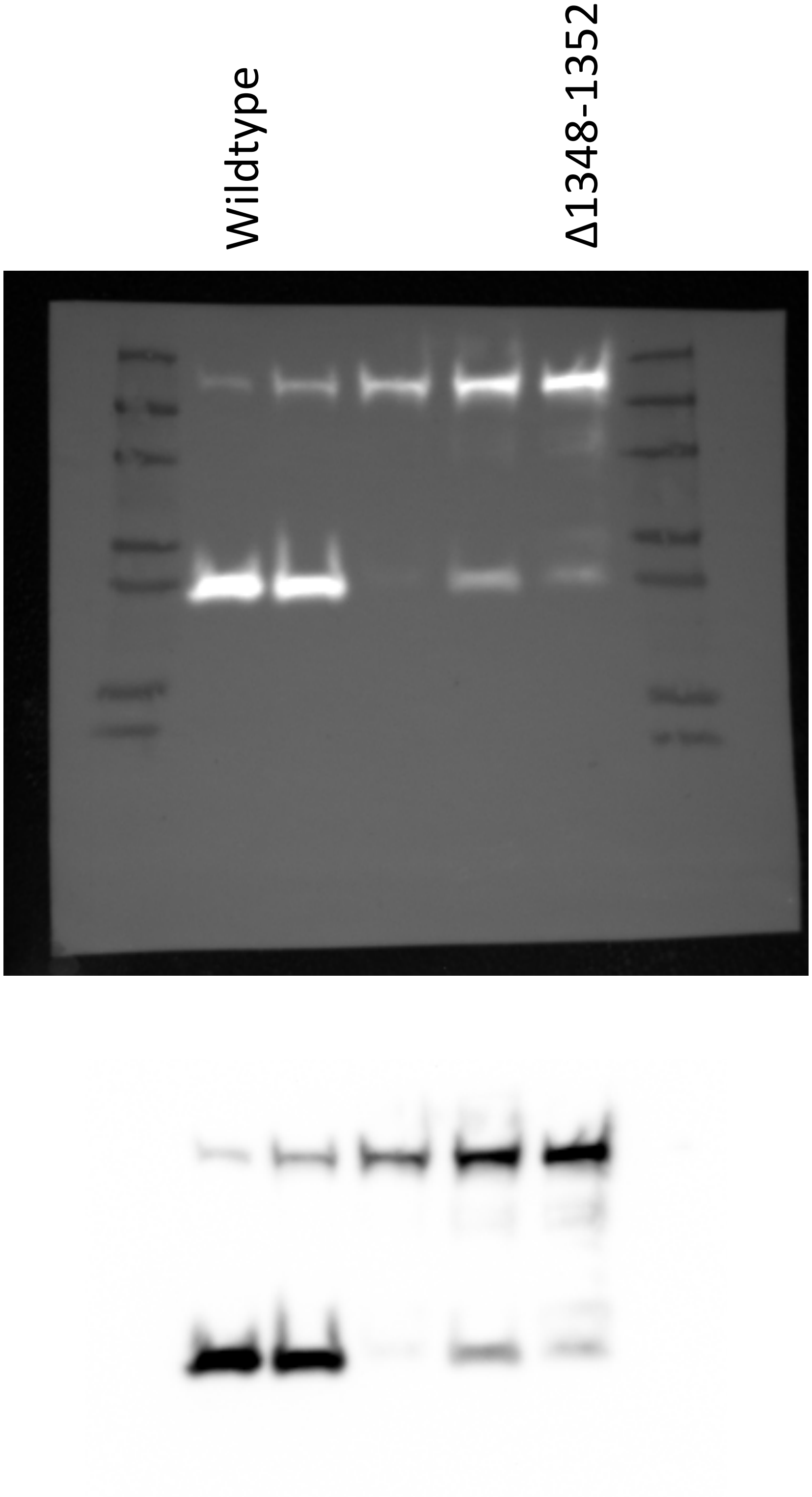

For figure 3B

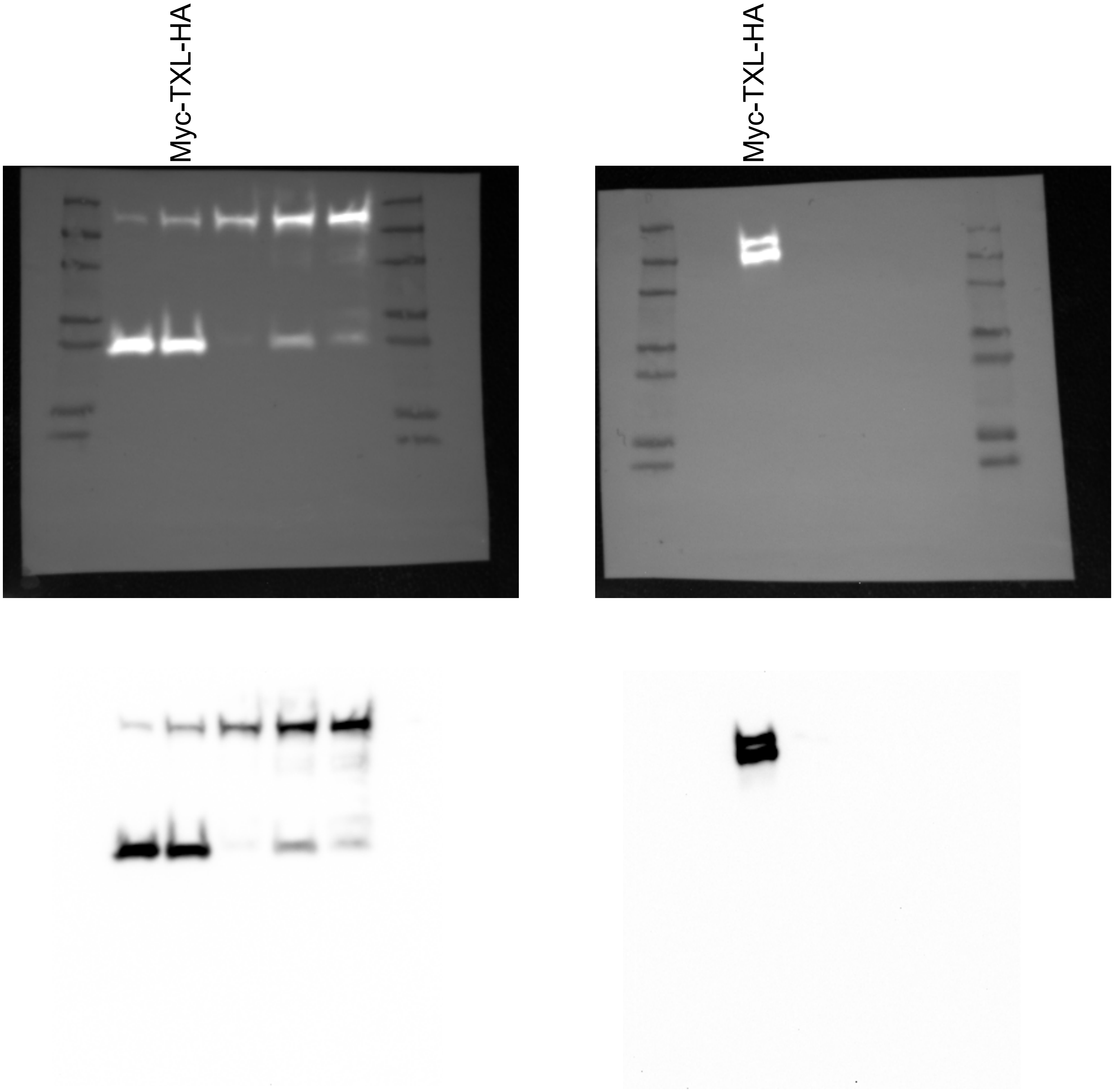

For figure 4B

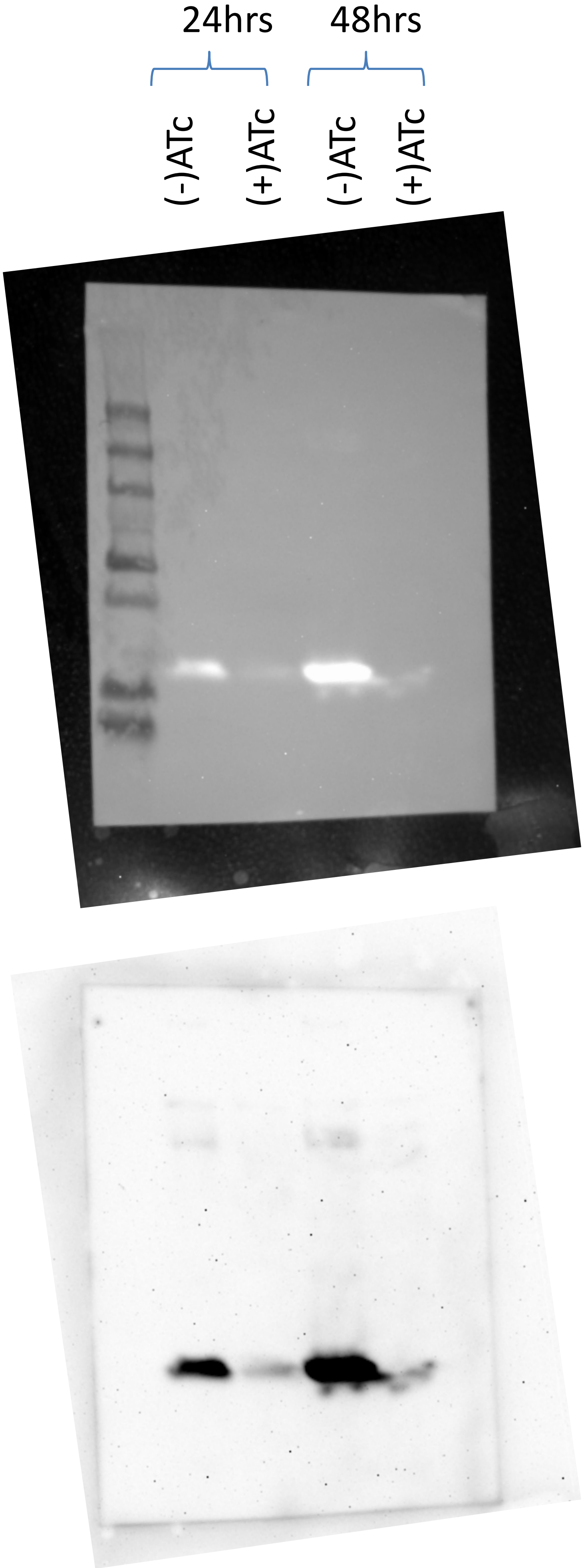

For figure 5B

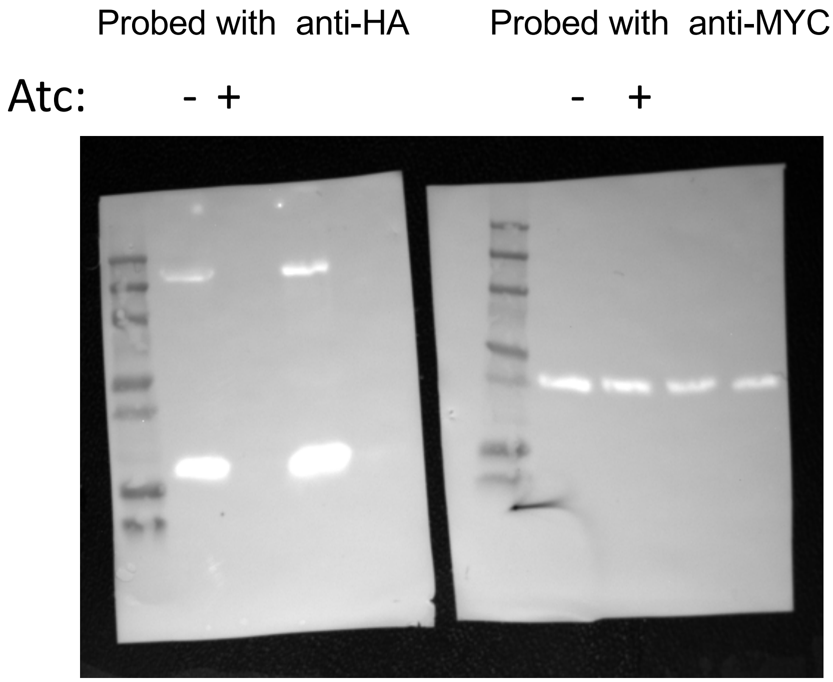

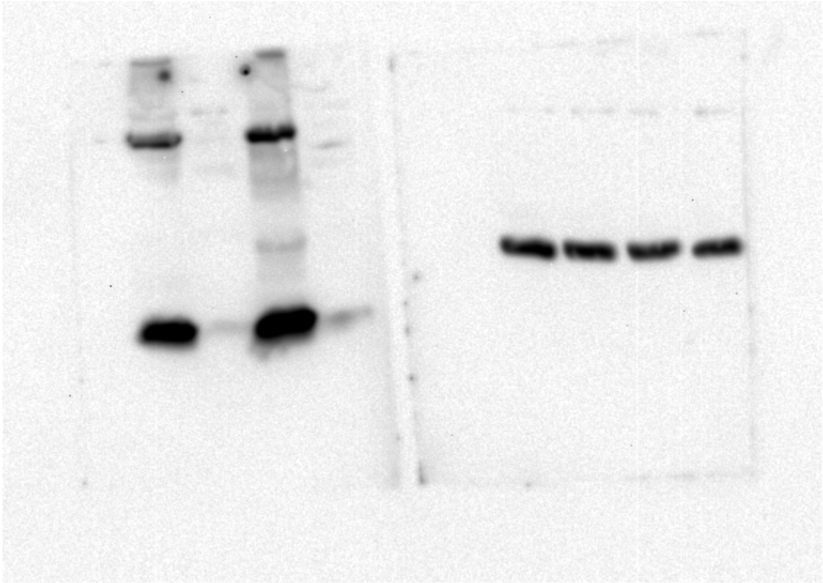

For figure 5E

Probed with anti-HA Probed with anti-MYC

1 2 3 1 2 3

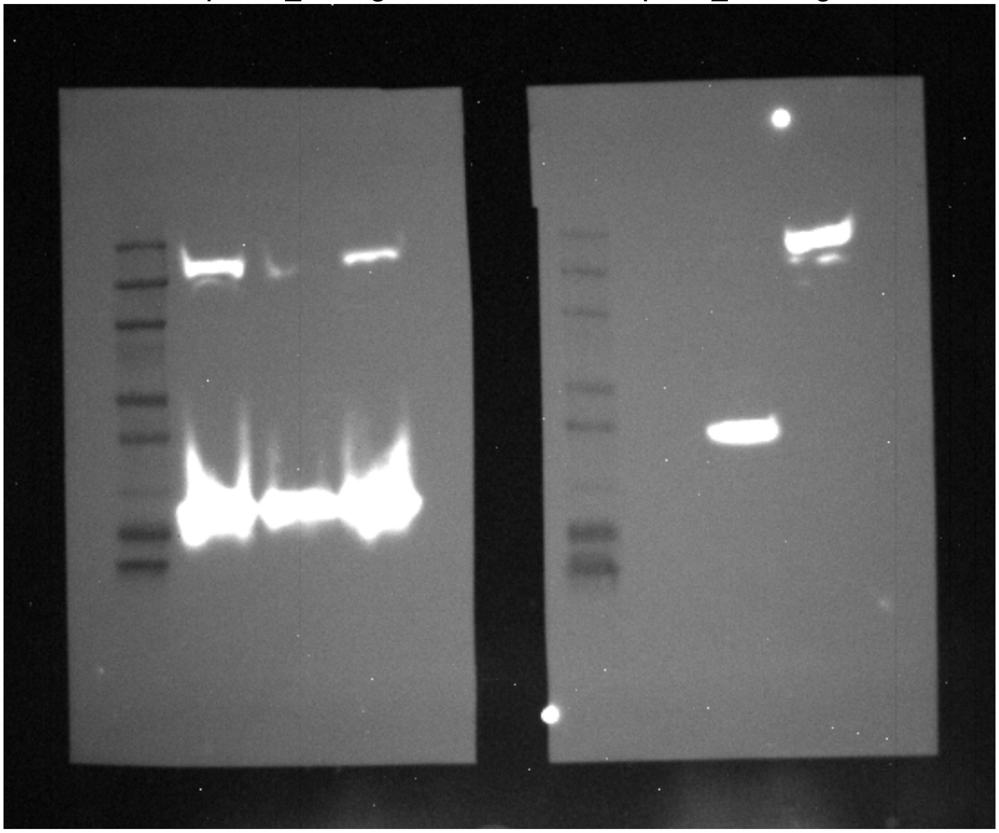

1: TATi GRA44(HA)

2: TATi GRA44(HA)
+GRA44(MYC)

3: TATi GRA44(HA)
+GRA44ΔTXL(MYC)

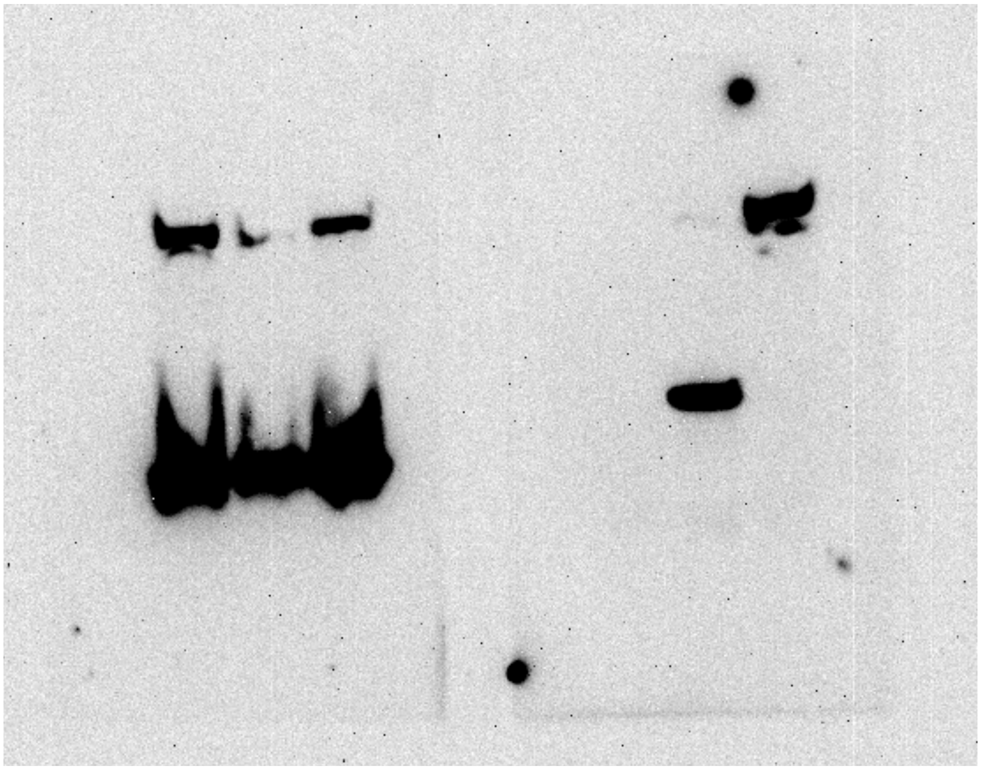

For figure 6

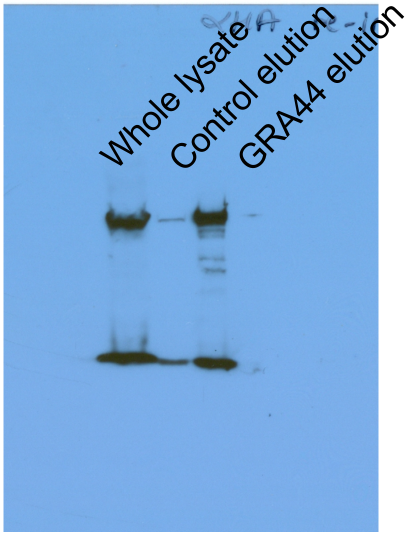

IP with anti HA, probed with HA

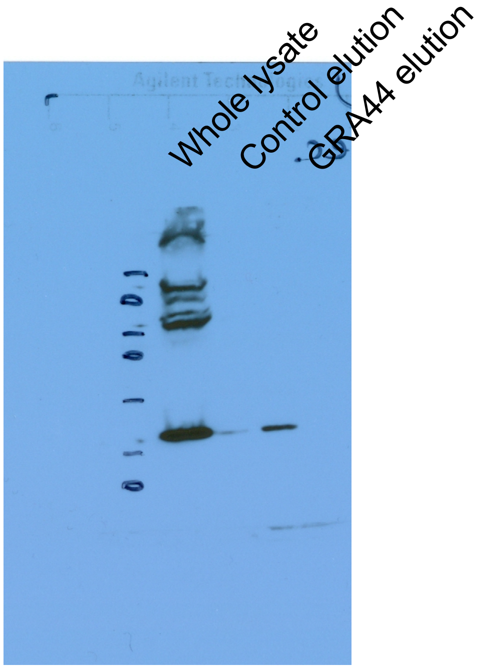

IP with anti HA, probed with anti myr1 C terminus

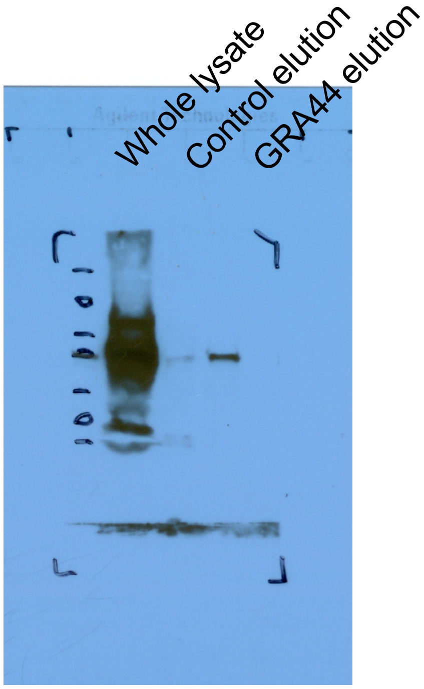

IP with anti HA, probed with anti myr1 N terminus
